# ER complex proteins are required for rhodopsin biosynthesis and photoreceptor survival in *Drosophila* and mice

**DOI:** 10.1038/s41418-019-0378-6

**Published:** 2019-07-01

**Authors:** Liangyao Xiong, Lin Zhang, Yeming Yang, Na Li, Wenjia Lai, Fengchao Wang, Xianjun Zhu, Tao Wang

**Affiliations:** 1 Peking University-Tsinghua University-National Institute of Biological Sciences Joint Graduate Program, School of Life Sciences, Peking University, Beijing, 100871 China; 2 National Institute of Biological Sciences, Beijing, 102206 China; 3 Sichuan Provincial Key Laboratory for Human Disease Gene Study, Sichuan Provincial People’s Hospital, University of Electronic Science and Technology of China, Chengdu, Sichuan 610072 China; 4 Southwest Jiaotong University, Chengdu, Sichuan China; 5 CAS Key Laboratory of Standardization and Measurement for Nanotechnology, CAS Center for Excellence in Nanoscience, National Center for Nanoscience and Technology, Beijing, 100190 China; 6 Tsinghua Institute of Multidisciplinary Biomedical Research, Tsinghua University, Beijing, 100871 China; 7 Chengdu Institute of Biology, Chinese Academy of Sciences, Chengdu, China; 8 Chinese Academy of Sciences Sichuan Translational Medicine Hospital, Chengdu, China; 9 Department of Ophthalmology, First People’s Hospital of Shangqiu, Shangqiu, Henan China

**Keywords:** Chaperones, Genetic interaction

## Abstract

Defective rhodopsin homeostasis is one of the major causes of retinal degeneration, including the disease Retinitis pigmentosa. To identify cellular factors required for the biosynthesis of rhodopsin, we performed a genome-wide genetic screen in *Drosophila* for mutants with reduced levels of rhodopsin. We isolated loss-of-function alleles in *endoplasmic reticulum membrane protein complex 3* (*emc3*), *emc5*, and *emc6*, each of which exhibited defective phototransduction and photoreceptor cell degeneration. EMC3, EMC5, and EMC6 were essential for rhodopsin synthesis independent of the ER associated degradation (ERAD) pathway, which eliminates misfolded proteins. We generated null mutations for all EMC subunits, and further demonstrated that different EMC subunits play roles in different cellular functions. Conditional knockout of the *Emc3* gene in mice led to mislocalization of rhodopsin protein and death of cone and rod photoreceptor cells. These data indicate conserved roles for EMC subunits in maintaining rhodopsin homeostasis and photoreceptor function, and suggest that retinal degeneration may also be caused by defects in early biosynthesis of rhodopsin.

## Introduction

Retinitis pigmentosa (RP) causes the gradual degeneration of rod and cone photoreceptors and is the most common form of hereditary retinal diseases [1, 2]. Mutations in the light receptor gene *rhodopsin* account for approximately 25% of all ADRP cases. Over 120 point mutations in the *rhodopsin* gene have been associated with ADRP, with most disrupting its trafficking from the endoplasmic reticulum (ER) to the plasma membrane [3]. Proper folding and targeting of rhodopsin is crucial for maintaining the structure, function, and homeostasis of photoreceptors [1].

Studying photoreceptor cells in *Drosophila* has provided insights into the folding and transport of rhodopsin protein [4, 5]. In flies, folding of rhodopsin is tightly regulated by multiple ER chaperons, including NINAA, calnexin, and Xport [6–8]. Without these chaperons the rhodopsin protein fails to properly mature, leading to retinal degeneration, as seen with mutations in rhodopsin itself [7, 9]. RanBP2 is a cyclophilin-related protein that acts as a chaperone for cone opsins, but many other chaperons required for rhodopsin folding are not well conserved in mammals [1, 10]. In fact, no mammalian homologs have been identified for any *Drosophila* gene involved in rhodopsin biogenesis in the ER that cause retinal degeneration when mutated.

The ER membrane protein complex (EMC) was first identified as a multi-protein transmembrane complex in a genetic screen for the accumulation of misfolded membrane proteins in yeast. Based on a high-content proteomics strategy, this complex was then found to interact with the ER-associated degradation (ERAD) pathway, suggesting a role in the biosynthesis of transmembrane proteins [11, 12]. Recently, it was shown that EMC functions as a transmembrane domain insertase, acting during the translation process to enable biogenesis of its client transmembrane proteins [13, 14]. EMC functions in multiple cellular processes, including autophagy, lipid transfer, viral infection, and lung development [15–22]. Mutation of the zebrafish *emc3* gene, *partial optokinetic response b* (*pob*), causes degeneration of long-wavelength photoreceptor cells, and *Drosophila* EMC3 is required for the stable production of rhodopsins [23, 24]. Moreover, a point mutation in the human *EMC1* gene has been associated with retinal dystrophy [25]. However, it has not yet been determined which steps in rhodopsin production require EMC components, and whether all EMC subunits are required for maintaining rhodopsin homeostasis and/or photoreceptor cell integrity.

In the present study, we conducted an ethyl methanesulfonate (EMS)-based genetic screen to isolate mutations that affect rhodopsin homeostasis. We identified mutations in 3 EMC subunits, *emc3*, *emc5*, and *emc6* that each reduced levels of the major rhodopsin Rh1, disrupted phototransduction, and caused gradual photoreceptor degeneration. We found that EMC function was independent of ERAD, most likely regulating rhodopsin levels at an earlier step in the biosynthetic process. Furthermore, knocking out the *Emc3* gene in mammalian rod photoreceptor cells led to dysregulated rhodopsin trafficking and retinal degeneration, as seen in flies, suggesting that EMC function is conserved in mammals. Our study highlights the different roles played by EMC proteins in rhodopsin biogenesis.

## Materials and methods

### EMS mutagenesis

The second chromosome of *ey-flp ninaE-Rh1-GFP;FTR40A* flies and the third chromosome of *ey-flp ninaE-Rh1-GFP;FTR82B* flies were isogenized, and young male flies were fed 25 mM ethyl methanesulfonate (EMS) (Sigma, St. Louis, MO) in 2% sucrose for 8 h. Mutagenized flies were mated immediately to *ey-flp ninaE-Rh1-GFP;GMR-hid CL FRT40A/Cyo hs-hid* or *ey-flp ninaE-Rh1-GFP; FRT82B GMR-hid CL/TM3 hs-hid* flies, and F1 progenies were screened for loss of GFP by performing the fluorescence deep pseudopupil (DPP) assays 1 day following eclosion [26]. Approximately 10,000 F1 flies of each genotype were screened. The *emc3*^*G7*^, *emc3*^*G10*^, and *emc5*^*G2*^ mutants were isolated by screening *ey-flp ninaE-Rh1-GFP;FTR40A* flies (looking for changes in Rh1 fluorescence); *emc6*^*N10*^ mutant flies were isolated by screening *ey-flp ninaE-Rh1-GFP;FTR82B* flies.

### Fly stocks

*CG3678*^*G4215*^ (*emc2B*), *CG32441*^*MB09773*^ (*emc10*), *ninaA*^*2*^, *ninaE*^*P332*^, *M(vas-int.Dm)ZH-2A;M(3xP3-RFP.attP)ZH-86Fb*, *M(vas-int.Dm)ZH-2A;M(3xP3-RFP.attP) ZH-51C*, *GMR-Gal4* and *dmppe*^*e02905*^ lines were obtained from the Bloomington Stock Center. The *emc4*^*RNAi*^ (*P{TRiP.HMC06642}attP40*) flies were obtained from the TsingHua Fly Center. The *emc2A*^*RNAi*^ and *emc5*^*RNAi*^ flies were generated using short-hairpin (sh) RNA system. The *emc1*, *emc2A*, *emc4*, *emc7*, *emc8/9*, and *hrd1* knockout lines were generated using Crispr/Cas9 technology. The *ey-flp ninaE-Rh1-GFP;FRT40A*, *ey-flp ninaE-Rh1-GFP;FRT42D*, *ey-flp ninaE-Rh1-GFP;FRT2A*, *ey-flp ninaE-Rh1-GFP;FTR82B*, *ey-flp ninaE-Rh1-GFP;GMR-hid CL FRT40A/Cyo hs-hid*, *ey-flp ninaE-Rh1-GFP;FRT42D GMR-hid CL/Cyo hs-hid*, *ey-flp ninaE-Rh1-GFP;GMR-hid CL FRT2A/TM3 hs-hid*, and *ey-flp ninaE-Rh1-GFP; FRT82B GMR-hid CL/TM3 hs-hid* lines were maintained in the laboratory of T. Wang. Flies were raised in a 12h-light-12h-dark cycle at 25 °C on standard corn food.

### Generation of the *emc1*^*1*^, *emc2A*^*1*^, *emc4*^*1*^, *emc7*^*1*^, *emc8/9*^*1*^, and *hrd1*^*1*^ mutant flies

The *emc1*, *emc2A*, *emc4*, *emc7*, *emc8/9*, and *hrd1* mutations were generated using the Cas9/sgRNA system, as described [27]. The recognition sequences of the guide RNAs were designed with tools available at the following website: http://www.flyrnai.org/crispr2/.

(*emc1*, sgRNA1: ACGCGTTCGTTGGTCGCGCG, sgRNA2: CCACCTACAGACACCCAGCG; *emc2A*, sgRNA1: GTTCCAAATATTCTCAGAAG, sgRNA2: TAGCGACATATTCACTTAGG; *emc4*, sgRNA: TCTTAAGAGGTCCCAGGGCA; *emc7*, sgRNA: GATAACCTCGCAGCTGACAA; *emc8/9*, sgRNA: CGACTACAAGGTCTCGGAGC; *hrd1*, sgRNA1: TACGGAGACCTGCTTGGCCT, sgRNA2: CTACGACATGCCCAGTACCT). The *(nos-Cas9)attP2* flies were used as the Cas9 source, and F1 progeny were screened by PCR and DNA sequencing to identify the mutations. The primers used for genomic PCR were as follows:

*emc1*-fwd, 5′-CGGAGTTGGAGCAGAGTC-3′

*emc1*-rev, 5′-GCGGTGTACAGATACATGCC-3′

*emc2A*-fwd, 5′-TTAGTGTGACCGTCGCTGG-3′

*emc2A*-rev, 5′-GTAAGCACATGGGCACGAG-3′

*emc4*-fwd, 5′-CTGGGCTACAATCCATCTGC-3′

*emc4*-rev, 5′-CCAAGAAGATACTAGACGACTG-3′

*emc7-*fwd, 5′-ATCATCGGCTGACAATTGG-3′

*emc7-*rev, 5′-GGGATATGCCACCTGCATAA-3′

*emc8/9*-fwd, 5′-GATGTGACCAGACTACTGC-3′

*emc8/9*-rev, 5′-ATGTATCGGACGCCTGTGAG-3′

*hrd1-*fwd, 5′-ATGCAGCTGCTCTTATCGTC-3′

*hrd1*-rev, 5′-CGTCTATTCCGCTGTCGTG-3′

### Generation of transgenic flies

The *emc3*, *emc5*, and *emc6* cDNAs were amplified from the cDNA clones LD37839, RE09053, and RE35789, respectively, which were obtained from the *Drosophila* Genomic Resource Center. To express *emc3* and *emc5* under control of the *ninaE* (*ninaE: neither inactivation nor afterpotential E*) promoter, the cDNAs were subcloned into the *pninaE-attB* vector between the *NotI* and *XbaI* sites, and the constructs were injected into *M(vas-int.Dm)ZH-2A;M(3xP3-RFP.attP)ZH-86Fb* embryos. To express *emc6* in photoreceptor cells, the cDNA was subcloned into the *pninaE-attB* vector between the *NotI* and *XbaI* sites, followed by injected into *M(vas-int.Dm)ZH-2A;M(3xP3-RFP.attP) ZH-51C*. The transformants were identified on the basis of eye color, and mini-white marker was eliminated by crossing to a Cre-expressing line.

To generate *emc2A*^*RNAi*^ and *emc5*^*RNAi*^ flies, the following 21-nt sequences were used to generate shRNA constructs, as described.

*emc2A*^*shRNA*^: 5′-GCAAGATTGCCATCCTTAAGG-3′

*emc5*^*shRNA*^: 5′-GCAGGAATGGAATAGCCTTCC-3′

Annealed oligo pairs were cloned into the VALIUM20 vector at the *NheI* and *EcoRI* sites [28]. Constructs were injected into *M(vas-int.Dm)ZH-2A;M(3xP3-RFP.attP)ZH-86Fb* embryo, and transformants were identified on the basis of eye color.

### ERG recordings

ERG recordings were performed as described [29]. Briefly, glass microelectrodes filled with Ringer’s solution were placed on the compound eye and the thorax of a fly. After a brief dark adaptation period, white-eyed flies were exposed to 5 s of orange light, followed by two rounds of 5 s of blue light, and two rounds of 5 s of orange light. The time between stimulations was 5 s. ERG signals were amplified with a Warner electrometer IE-210, and recorded with a MacLab/4 s analog-to-digital converter and the clampelx 10.2 program (Warner Instruments, Hamden, USA).

### Antibodies

Primary antibodies used for western blotting are listed below: Tubulin (mouse, 1:2000, Developmental Studies Hybridoma Bank), ß-Actin (mouse, 1:2000, Santa Cruz), Rh1 (mouse, 1:2000, Developmental Studies Hybridoma Bank), TRPL (rabbit, 1:500, Fisher, Waltham, USA) [30], TRP (rabbit, 1:2000) [29], INAD(rat, 1:2000) [29]. The TRP and INAD antibodies were a gift form Dr. C. Montell. Secondary antibodies were bought from LI-COR Biosciences, which included IRDye 680 goat anti-mouse-IgG, IRDye 800 goat anti-rabbit-IgG, and IRDye 800 goat anti-rat-IgG antibodies (1:10000).

Primary antibodies used for staining are listed below: Rh1 (mouse, 1:200, Developmental Studies Hybridoma Bank), Cnx99A (mouse, 1:20, Developmental Studies Hybridoma Bank), GM130 (rabbit, 1:200, #ab30637, Abcam) GFP (rabbit, 1:200, Invitrogen), RFP (rat, 1:200, ChromoTek), EMC3 (rabbit, #ab175537, Abcam), NaK ATPase (mouse, #NA163540, Thermo Scientific), M-Opsin opsin (AB5405, Millipore, MA, USA), Rho 1D4 monoclonal antibody against rhodopsin was a gift from Dr. Robert Molday, University of British Columbia, Canada. Secondary antibodies were bought from Invitrogen (anti mouse, rabbit or rat IgG labeled with Alexa Fluor 488, Alexa Fluor 568, Alexa Fluor 594, Alexa Fluor 647, or Alexa Fluor 488), 1:500 dilution.

### Immunostaining

Retina were dissected and fixed in 4% freshly made paraformaldehyde in phosphate buffer for 2 h on ice, then dehydrated in a series of ethanol dilutions (10, 25, 40, 55, 75, 90, 100%), 30 min each. Samples were then treated in acetone solution, and embedded in LR white resin (Sigma, St Louis, MO). Sections (1 μm) were then prepared for immunostaining. Samples were blocked using PBST (0.3% Triton X-100) with 5% goat serum for 1 h, then incubated with primary antibodies at 4 °C overnight. This was followed by incubation with secondary antibodies for 1 h. Samples were examined and images were recorded using a Nikon A1-R confocal microscope, and further processed using Photoshop CC2017 software.

### Co-immunoprecipitation

Flies were kept in a 12h-light-12h-dark cycle at 25 °C. Approximately 200 flies were collected 5 days after eclosion. The flies were frozen in liquid nitrogen to dissociate the head from the body. Heads were then sieved out and lysed using lysis buffer (50 mM Tris-HCl, pH 7.5, 150 mM NaCl, 1% NP40, 1 × proteasome inhibitor) at 4 °C overnight. They were then incubated with RFP agarose beads (Chromotek) for 2 h at 4 °C. After several washes, the beads were boiled in SDS loading buffer, and supernatant was loaded onto a gel. Sliver staining (Thermo Fisher Scientific) was performed and bands on the gel were cut out for further mass spectra analysis.

### Proteomic analysis of the fly retina

Flies were maintained in a 12 h-light/12h-dark cycle at 25 °C. Sixty female flies of each genotype were collected 5 days after eclosion. For each sample, 30 pairs of retinas were dissected in cold phosphate buffer. Two samples were generated for each genotype. Protein extraction was performed as described with several modifications [31]. Briefly, tissues were boiled and sonicated in lysis buffer (6 M guanidinium hydrochloride, 20 mM tris (2-carboxyethyl) phosphine, 50 mM chloroacetamide, 50 mM Tris, pH 8.5), followed by 20 min 18,000 g centrifugation to collect supernatant. About 50 µg protein for each sample was digested with LysC (Promega Corporation, Madison, USA) in an enzyme/protein ratio of 1:100 (w/w) for 4 h. The sample was further digested with trypsin (Promega Corporation, Madison, USA) in an enzyme/protein ratio of 1:50 (w/w) overnight at 37 °C. Finally, the resulting peptides were acidified with trifluoroacetic acid and desalted by C18 column (3 M, Bracknell, UK) as described [32]. The peptide samples were labeled by TMT 10plex™ Isobaric Label Reagent Set label kit (Thermo, USA) according to the instruction. The mixed peptides from 10 samples (two replicates 50 µg each) were fractionated using a reversed-phase C18 column (3 M, Bracknell, UK) as described with some modifications [33]. Briefly, 8 fractions of peptide were eluted with acetonitrile step gradients (7.5, 10, 12.5, 15, 17.5, 20, 22.5, and 50%, pH 10). Then, the 8 fractions were dried in a vacuum centrifuge and stored at −80 °C until LC-MS/MS analysis.

About 2 µg of each high-pH fractionated peptides samples were separated on an in-house packed 75-μm ID × 50 cm capillary column with 2.5 μm Venusil C18 beads (Agela Technologies, China) using an EASY-nLC 1000 system (Thermo Scientific, Odense, Denmark). The column temperature was maintained at 42 °C using an integrated column oven (PRSO-V1, Sonation GmbH, Biberach, Germany). The flow rate was set to 200 nL/min, and a total 240 min or 320 min gradient from 2 to 27% acetonitrile was used. Raw data was collected on Q Exactive mass spectrometer (Thermo Scientific, Bremen, Germany) using Thermo Xcalibur (2.0) control software with a data-dependent MS/MS scans (TopN = 15). MS1 spectra was measured at a resolution of 70,000 at m/z 200, and full scan target was 3 × 10^6^ with a maximum injection time of 50 ms. Mass range was set to 320–1500. The maximum ion injection time for the MS/MS scan was 100 ms with a resolution of 35,000 at *m/z* 200, and target value for fragment scans was set at 1 × 10^5^. Isolation width was set at 1.2 *m/z* and normalized collision energy was set at 32. Each fraction was analyzed in twice.

All raw LC−MS/MS data were submitted to Proteome Discoverer (2.2 version, Thermo Science) for TMT quantitation and database analysis using SequestHT. Data were searched against the *Drosophila Melanogaster* (Fruit fly) Swiss-Prot database (UP000000803, 21,922 sequences) combined with a common contaminants database (247 entries), using default processing and consensus workflow for MS2 TMT quantification method. The standard searching parameters were used: a 10 ppm MS1 error tolerance was used, with 0.02 Da error used for MS2 product ions on the Q Exactive. Trypsin was set as the enzyme, allowing for 2 missed cleavages. Methionine oxidation was set as a variable modification, and carbamidomethyl on cysteines, TMT6 plex on lysine and peptide N-terminus were set as static modifications for all searches. FDR was set to 0.01 for peptide database search.

### Cell transfection

S2 cells were transfected by adding 5 µg of plasmid mixed with 2 µl VigoFect agent (Vigorous Biotechnology) into the cell media. Twenty-four hours following transfection, cells were suspended and adhered to a poly-lysine treated coverslip for 1 h and then fixed for 0.5 h in freshly made 4% paraformaldehyde in phosphate buffer. Samples were blocked using PBST (0.3% Triton X-100) with 5% goat serum for 1 h, then incubated with primary antibodies for 1 h. After washing with phosphate buffer, cells were incubated with secondary antibodies for 1 h. Samples were then imaged using a Nikon A1-R confocal microscope. Images were further processed using Photoshop CC2017 software.

### Generation of *Emc3* knockout mice

All mouse study protocols were approved by the Animal Care and Use Committee of Sichuan Provincial People’s Hospital. All experimental procedures were carried out in accordance with the approved study protocols and relevant regulations. Mice were raised in a 12h-light-12h-dark cycle. The *Emc3* conditional knockout allele was generated using the CRISPR/Cas9 system using gRNA sequence CCTTTGTGAAAACCTGTGTGGCA. A DNA template containing two loxp sites flanking exon 2 of *Emc3* was used to guide DNA repair: ccttcctcagaaacctttatttgttcagagcccagtcactctggaagactgccttggatccATAACTTCGTATAATGTATGCTATACGAAGTTATggctgtgaaaacctgtgtggcaagttgaatatgtttcattgatttttagggtttttggaaagtttccacacccagcttttgccaaccaggtcctcccttatgtctgagtgggccttctactttacgcttgctttctttgcgtttcacattttaacatctctttgtcttgtgtttttcagTCAGGTCCTAATTCGAAGCAGAGTCCTCAGGGAAAATGGAAAATACATTCCCAAGCAGgtactcactgatattttatttagaggctccaccattcacctgtaagggcagtaaaaacctaaatgtttttttataagaggatgccagagaaaactggaggtggccatccagttattaagctgcagcATAACTTCGTATAATGTATGCTATACGAAGTTATgaattcggtggtggtgtttgctgtcatttgccctcacccttcttaggaaattgtttttaaatgtgactca. Loxp sequences were underlined. Intronic sequences were in lower case letters. Exon 2 was in upper case letters. C57BL/6 J mice were used for embryo injection. Positive founder mice were screened using PCR and DNA sequencing. Primer pair Emc3-screen-F1 and Emc3-screen-R1 was used to identify upstream loxp sequence. Emc3-screen-F1: CCCAGGACGGGGATTATAGTGGTGTG; Emc3-screen-R1: ACTTGCCACACAGGTTTTCACAGCC. A second primer pair Emc3-screen-F1 and Emc3-screen-R2 were used to identify downstream loxp sequence. Emc3-screen-F1: CCCAGGACGGGGATTATAGTGGTGTG; Emc3-screen-R2: CAAATGACAGCAAACACCACCACCG. Cone-specific knockout mice were generated by crossing *Emc3*^*loxp/+*^ to HRGP-Cre mice [34]. The *rd1* mutation was bred out before the experiment. *Emc3*^*loxp/+*^ mice were crossed to the broadly expressed inducible CRE CAG-Cre-ER mice (stock Tg(CAG-cre/Esr1*) 5Amc/J, https://www.jax.org/strain/004453) to generate inducible adult knockout mice [35].

### Tamoxifen treatment

A total of 100 mg of tamoxifen salt (Sigma, St Louis, MO, USA) was dissolved in 10 ml of ethanol as a stock solution. On the day of injection, a 1 mg/ml working solution was prepared by mixing 10 mg/ml stock solution with corn oil (Sigma-Aldrich, St Louis, MO, USA) and mixed well. On P20, mutant and control mice were intraperitoneally injected with a daily dosage of 25 mg/kg body weight for 3 days [35].

### Genotyping by PCR

Genomic DNA extracted from mouse tails was amplified by PCR using the following primer pairs: EMC3-Seq-F1, TGTCTCCCGTCAAATCCAGAAAGG; EMC3-Seq-R1, ATGTGAAACGCAAAGAAAGCAAGC. Amplification was performed using an Invitrogen Platinum SuperFi PCR master mix (Catalog # 12358–050, Invitrogen, Waltham, MA, USA). The first cycle consisted of 95 °C for 2 min, followed by 33 cycles of 94 °C for 15 s, 58 °C for 20 s, and 72 °C for 30 sec. Cre was genotyped using generic Cre primers: Cre-F, TGCCACGACCAAGTGACAGCAATG, and Cre-R, ACCAGAGACGCAAATCCATCGCTC).

### Immunostaining of mouse retinas

For immunohistochemistry, enucleated eyes were removed, marked at the nasal side for orientation, and fixed for 3 h in 4% paraformaldehyde in 100 mM phosphate buffer (PB) (pH 7.4) and then cryoprotected in 30% sucrose. Tissues were embedded in optimal cutting temperature solution (OCT) and frozen on dry ice for sectioning. Sections (10 micron) were blocked and permeabilized with 10% normal goat serum and 0.1% Triton X-100 in phosphate buffer for 60 min. Labelling with various antibodies was performed as previously described [36]. Primary antibodies were diluted in phosphate buffer containing 5% normal goat serum and 0.1% Triton X-100. Sections were incubated with primary antibodies overnight. Then, the sections were washed with PB three times and labelled for 1 h with secondary antibody and counterstained with DAPI.

Quantification of mislocalized M-Opsin in the cell bodies of cone cells was performed as follows. P30 and P50 cross-sections of retinas were stained using M-Opsin opsin and DAPI, and higher-magnification images were captured on a Leica SP8 confocal microscope. The number of cones in which M-Opsin mislocalized to the inner segment and to the cell body was counted. Slides were photographed on a Zeiss LSM 800 confocal scanning microscope.

### Histology and measurement of outer nuclear layer of the mouse retinas

For H&E staining, eyes from WT and knockout (KO) mice were removed, marked on the nasal side for orientation, and fixed overnight in 1.22% glutaraldehyde and 0.8% paraformaldehyde in 0.08 M phosphate buffer. They were embedded in paraffin, and cut into 5-µm sections. H&E stained sections were used to count the rows of photoreceptors in the outer nuclear layer [37]. Three measurements of the outer nuclear layer were taken every 200 μm from the optic nerve and averaged. The optic nerve was designated as 0 µm.

## Results

### EMC3, EMC5, and EMC6 are required for rhodopsin expression

**Fig. 1:**
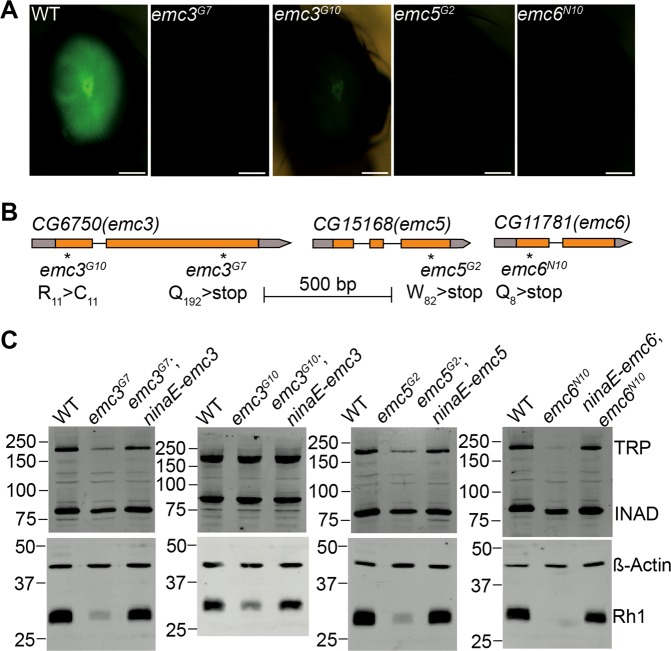
Rhodopsin biosynthesis defects in the *emc3*, *emc5*, and *emc6* mutants. **a** Isolation of the *emc3*^*G7*^, *emc3*^*G10*^, *emc5*^*G2*^, and *emc6*^*N10*^ mutations via a forward genetic screen. Rh1-GFP fluorescence in the deep pseudopupil of 1-day-old wild-type (*ey-flp rh1-GFP;FRT40A/GMR-hid CL FRT40A*), *emc3*^*G7*^ (*ey-flp rh1-GFP;emc3*^*G7*^ *FRT40A/GMR-hid CL FRT40A*), *emc3*^*G10*^ (*ey-flp rh1-GFP;emc3*^*G10*^ *FRT40A/GMR-hid CL FRT40A*), *emc5*^*G2*^ (*ey-flp rh1-GFP;emc5*^*G2*^ *FRT40A/GMR-hid CL FRT40A*), and *emc6*^*N10*^ (*ey-flp rh1-GFP; FRT82B emc6*^*N10*^*/FRT82B GMR-hid CL*) flies are shown. Scale bar is 100 μm. **b** The *emc3*, *emc5*, and *emc6* loci and mutations associated with the *emc3*^*G7*^, *emc3*^*G10*^, *emc5*^*G2*^, and *emc6*^*N10*^ alleles. **c** Rhodopsin levels were reduced in *emc3*^*G7*^, *emc3*^*G10*^, *emc5*^*G2*^, and *emc6*^*N10*^ flies, and were restored by *ninaE-emc3*, *ninaE-emc5*, and *ninaE-emc6* transgenes, respectively. Protein extracts from 1/4 head of each genotype (1 d after eclosion) were loaded and probed with antibodies against Rh1, ß-actin, TRP, and INAD

To identify genes involved in rhodopsin homeostasis, we performed EMS mutagenesis and screened chromosome 2 and 3 using the *Rh1::GFP ey-flp/hid* system, which utilizes the FLP-FRT recombination system to generate eyes homozygous for mutations, while using GFP-tagged Rh1 to track photoreceptor cell integrity [26, 38]. Among the ~20 mutants which displayed low GFP fluorescence (Figure S1), four were mutations in genes encoding different EMC components (Fig. 1a). The *emc3*^*G7*^ and *emc3*^*G10*^ mutations are two new *emc3* alleles, containing nonsense and missense mutation, respectively. One new (mutant *emc5*^*G2*^) introduces a premature stop codon in the CG15168 gene, which encodes an EMC5 homologue. The *emc6*^*N10*^ allele introduces a nonsense mutation in the CG11781 gene, which encodes an EMC6 homologue (Fig. 1b and Figure S2).

We confirmed via western blotting that endogenous Rh1 protein levels were greatly reduced in *emc3*^*G7*^, *emc3*^*G10*^, *emc5*^*G2*^, and *emc6*^*N10*^ mutant animals. Moreover, restoration of EMC3, EMC5, and EMC6 protein in *emc3*^*G7*^, *emc3*^*G10*^, *emc5*^*G2*^, and *emc6*^*N10*^ photoreceptor cells via the *ninaE* promoter, completely restored Rh1 levels (Fig. 1c and Figure S3). These results indicate that EMC is essential for rhodopsin biosynthesis. To analyse the specificity of EMC3, EMC5, and EMC6, we measured levels of TRP and INAD, exclusively function in the phototransduction cascade. INAD, a cytosolic scaffold protein has little changes in all 3 mutants, indicating the integrity of photoreceptor cells. However, TRP protein levels were moderately reduced in *emc5*^*G2*^ and *emc3*^*G7*^ mutants, but dramatically downregulated in *emc6*^*N10*^ mutants (Fig. 1c and Figure S3). This suggests that EMC3, EMC5, and EMC6 are involved in regulating the levels of other transmembrane proteins in photoreceptor cells, albeit to different extents.

### Loss of Rh1 in *emc* mutants disrupted phototransduction in flies

**Fig. 2:**
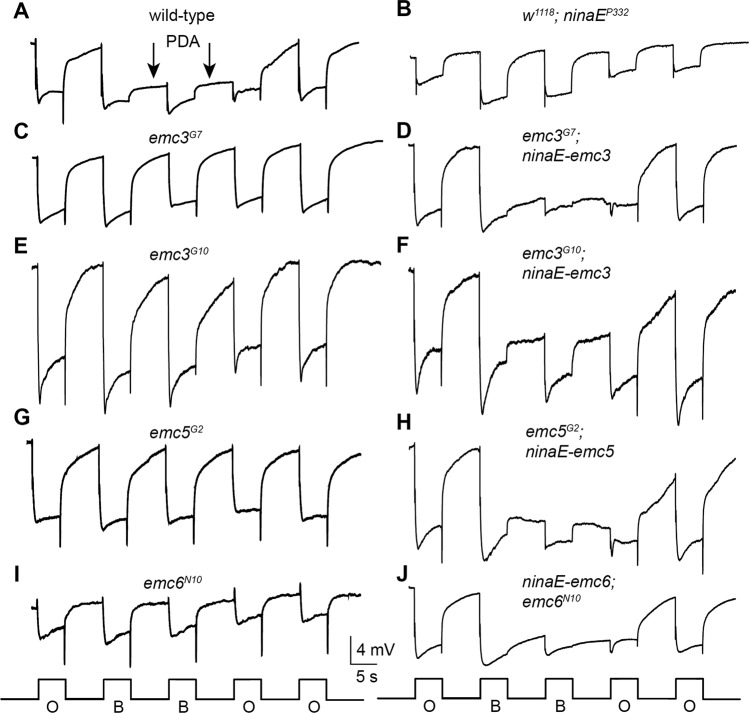
Loss of PDA in *emc3*, *emc5*, and *emc6* mutants. **a** ERG recordings in wild-type flies (*ey-flp rh1-GFP;FRT40A/GMR-hid CL FRT40A*) showed that PDA was induced by blue light and terminated by orange light (arrows). **b** PDA was eliminated in *ninaE*^*P332*^ flies and in **c** *emc3*^*G7*^, **e** *emc3*^*G10*^, **g** *emc5*^*G2*^, and **i** *emc6*^*N10*^ flies. PDA was restored in (**d**) *emc3*^*G7*^*;ninaE-emc3*, **f** *emc3*^*G10*^*;ninaE-emc3*, **h** *emc5*^*G2*^*;ninaE-emc5*, and **j** *ninaE-emc6;emc6*^*N10*^ flies. Flies ~1day after eclosion were dark-adapted for 2 min and subsequently exposed to 5-s pulses of orange (**O**) or blue (**B**) light. At least 10 flies of each genotype were tested

Since the major form of rhodopsin Rh1 is essential for photoreceptor function, we asked whether phototransduction was disrupted in *emc* mutants. Electroretinogram (ERG) is an extracellular recording that measures the summed light responses of all retinal cells (Fig. 2a). After wild-type flies were exposed to blue light, a prolonged depolarization afterpotential (PDA) was detected, reflecting an excessive accumulation of active Rh1 (Fig. 2a) [39]. In mutants with reduced levels of Rh1, PDA was not produced (Fig. 2b) [40, 41]. Similarly, PDA was not detected in *emc3*^*G7*^, *emc3*^*G10*^, *emc5*^*G2*^, and *emc6*^*N10*^ mutants. Restoring EMC3, EMC5, and EMC6 protein levels rescued the PDA response in *emc3*^*G7*^, *emc3*^*G10*^, *emc5*^*G2*^, and *emc6*^*N10*^ mutants, respectively (Figs. 2c–j). Importantly, the ERG phenotypes of *emc3*^*G7*^, *emc3*^*G10*^, *emc5*^*G2*^, and *emc6*^*N10*^ flies were indistinguishable from *ninaE* mutants, but different from *trp* mutants [42, 43]. This suggests that EMC affects phototransduction by regulating rhodopsin production.

### Rhodopsin is properly folded and is not degraded through the ERAD pathway in *emc* mutants

**Fig. 3:**
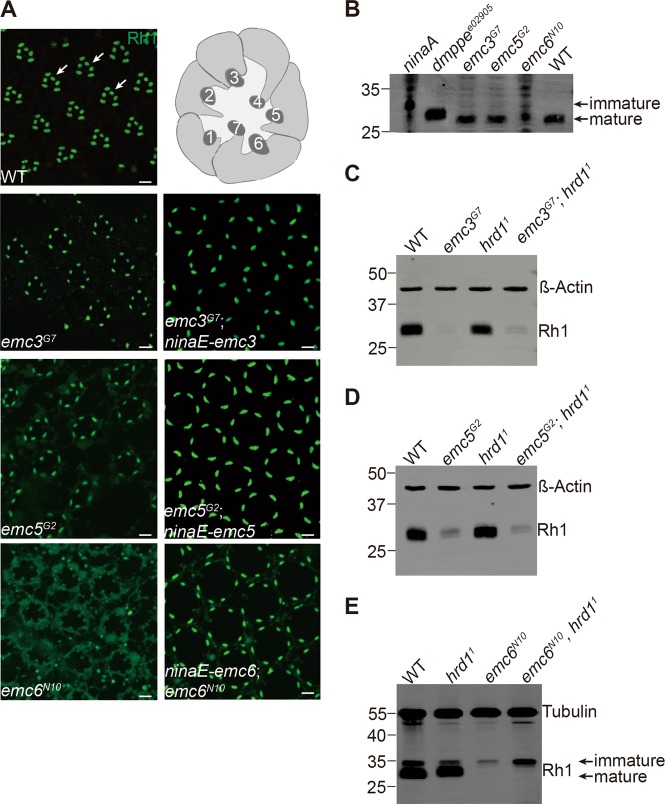
EMC3, EMC5, and EMC6 were not required for maturation of the Rh1 protein. **a** Rh1 localized to the rhabdomere region in *emc3*, *emc5*, and *emc6* mutant photoreceptor cells. Tangential resin-embedded retina sections of compound eyes from ~1-day-old wt, *emc3*^*G7*^, *emc3*^*G7*^*;ninaE-emc3*, *emc5*^*G2*^, *emc5*^*G2*^*;ninaE-emc5, emc6*^*N10*^, and *ninaE-emc6;emc6*^*N10*^ flies were labeled using antibodies against Rh1. Rhabdomeres are indicated by arrows. Scale bar is 2 μm. Illustration of photoreceptor cells within a single ommatidium is on the top right corner, and rhabdomeres of R1-R7 photoreceptor cells are indicated by numbers. **b** Deglycosylation of Rh1 is normal in *emc3*, *emc5*, and *emc6* mutants. Head extracts prepared from 1-day-old wild-type, *ninaA*, *dmppe*^*e02905*^, *emc3*^*G7*^, *emc5*^*G2*^, and *emc6*^*N10*^ flies were probed with antibodies against Rh1, and mature and immature bands are indicated. Extracts from *ninaA* and *emc6*^*N10*^ flies (10 heads), *emc3*^*G7*^ and *emc5*^*G2*^ flies (5 heads), and wild-type and *dmppe*^*e02905*^ flies (0.25 of a head) were loaded. **c**–**e** Similar Rh1 levels were seen in (**c**) *emc3*^*G7*^ and *emc3*^*G7*^*;hrd1*^*1*^ flies (*ey-flp rh1-GFP;emc3*^*G7*^ *FRT40A/GMR-hid CL FRT40A;FRT82B hrd1*^*1*^*/FRT82B GMR-hid CL*), **d** *emc5*^*G2*^ and *emc5*^*G2*^*;hrd1*^*1*^ flies (*ey-flp rh1-GFP;emc5*^*G2*^ *FRT40A/GMR-hid CL FRT40A;FRT82B hrd1*^*1*^*/FRT82B GMR-hid CL*), and **e** *emc6*^*N10*^ and *emc6*^*N10*^ *hrd1*^*1*^ (*ey-flp rh1-GFP; FRT82B emc6*^*N10*^ *hrd1*^*1*^*/FRT82B GMR-hid CL*) flies. Protein extracts from 0.25 of a head from ~1-day-old flies were loaded

To determine which step of the rhodopsin biosynthetic process requires EMC, we examined the localization of endogenous Rh1 in resin-embedded retina sections from *emc* mutants. As with wild-type flies, the majority of Rh1 localized to the rhabdomere (the tightly-packed microvilli in which phototransduction occurs) in *emc3*^*G7*^ and *emc5*^*G2*^ flies, although the Rh1 signal was weak compared to rescued flies. This indicates that Rh1 trafficking was normal in both *emc3* and *emc5* mutants (Fig. 3a). However, Rh1 levels were much lower in *emc6*^*N10*^ mutants, and Rh1 was detected in the cell body (Fig. 3a). Restoring EMC3 and EMC5 protein levels rescued Rh1 signal in *emc3*^*G7*^ and *emc5*^*G2*^ mutants, respectively, and EMC6 restoration rescued both Rh1 signal and localization in *emc6*^*N10*^ flies, albeit not fully mimic wild-type morphology, which may due to different sectioning angle and depth. During biosynthesis, Rh1 is transiently glycosylated in the ER lumen, and this modification is progressively removed as it is transported to the rhabdomere. If Rh1 quality control or trafficking is blocked, such as in *ninaA* [6] or *dmppe*^*e02905*^ [44] mutant flies, immature Rh1, which has a higher molecular weight, can be detected [38, 45]. The major Rh1 protein isolated from *emc3*^*G7*^ and *emc5*^*G2*^ flies was about the same size as in wild-type flies. In *emc6*^*N10*^ flies, the major Rh1 band was the mature form, but immature Rh1 was also detected (Fig. 3b). This is consistent with the detection of Rh1 in the cell body, and suggests that EMC6 may have distinct functions.

It has been shown that EMCs functionally interacts with the ERAD pathway, which degrades unassembled polypeptides on the ER [11]. Thus, it is possible that the massive degradation of newly synthesized rhodopsin is responsible for the reduced levels of Rh1 in *emc* mutants. To test this hypothesis, we generated a deletion mutation for the *hrd1* gene (Figure S4), which encodes the major E3 ligase in the ERAD pathway [46]. Eliminating *hrd1* did not affect Rh1 levels, and more importantly, loss of *hrd1* failed to rescue Rh1 levels in *emc3*^*G7*^, *emc5*^*G2*^, and *emc6*^*N10*^ mutants (Figs. 3c–e). These data suggest that EMCs regulate rhodopsin biosynthesis independent of the ERAD pathway.

### Mutating EMC components caused different phenotypes

Since the *emc5*, *emc3*, and *emc6* mutants exhibited similar phenotypes, we assumed that they function through forming the EMC complex. We expressed mCherry-tagged EMC5 in photoreceptor cells, and performed an immunoprecipitation assay with the RFP-Trap followed by mass spectra identification (Figure S5A). We found that most EMC subunits, including EMC3, EMC4, EMC7, EMC8/9, and EMC10, were pulled down (Figure S5B and Supplementary data). This agreed with other mass spectra data indicating that different EMC subunits interact with one another [11, 19].

**Fig. 4:**
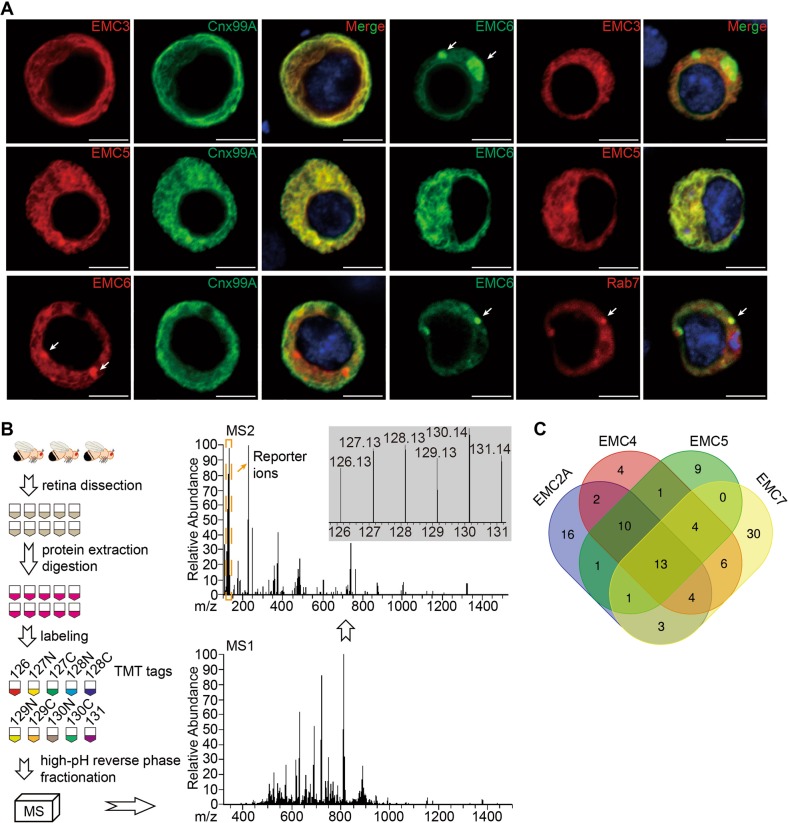
Interaction among EMC subunits. **a** S2 cells were transiently transfected with *emc3-RFP*, *emc5-mCherry*, or *emc6-GFP*, or co-transfected with *emc3-RFP/emc6-GFP*, *emc5-mCherry/emc6-GFP*, *emc6-GFP/RFP-rab7*. EMC6 puncta are indicated by arrows. Scale bar is 5 μm. **b** Flowchart of Tandem Mass Tags Labeling coupled with LC-MS/MS for the comparative analysis of protein levels in retina of *emc2* (*GMR-Gal4/UAS-emc2A*^*RNAi*^), *emc4* (*GMR-Gal4/UAS-emc4*^*RNAi*^), *emc5* (*GMR-Gal4/UAS-emc5*^*RNAi*^), and *emc7* (*FRT42D emc7*^*1*^) mutant flies, as well as wild-type (*GMR-gal4*) flies. **c** Venn diagrams showing the overlap of proteins that were downregulated > 50% among the four analyzed genotypes: *emc2*, *emc4*, *emc5*, and *emc7*

We next characterized the cellular localization of EMC3, EMC5, and EMC6 in S2 cells. In contrast to EMC5 and EMC3 which co-localized with the ER marker, Cnx99A (calnexin), EMC6 primarily localized to intracellular vesicles that lacked ER markers (Fig. 4a). However, when EMC5 and EMC6 were co-expressed in S2 cells, EMC6 completely colocalized with EMC5, which was not observed when EMC3 and EMC6 were co-expressed (Fig. 4a). To further investigated the compartments in which EMC6 may function independent of the EMC complex in ER, we examined the localization of EMC6 with markers of different intracellular compartments. EMC6 partially co-localized with Rab7, a late endosome marker, but not with Rab5, Lamp1, GM130, or Golgin245, suggesting that EMC6 may also function in the late endosome independent of the EMC complex (Fig. 4a and Figure S6). These results suggest that in addition to its functions as a subunit of the EMC complex, EMC6 may also perform distinct functions.

Although EMC subunits form a complex, our genome-wide screen for genes that disrupt rhodopsin biosynthesis only identified *emc3*, *emc5*, and *emc6*. We therefore asked if other EMC components also regulated biosynthesis of rhodopsin. Using the CRISPR/Cas9 system, we generated null mutations for all genes encoding EMC components, namely *CG2943* (*emc1*), *CG17556* (*emc2A*), *CG11137* (*emc4*), *CG8397* (*emc7*), and *CG3501* (*emc8/9*) (Figure S4). We also obtained the transposon insertion lines *CG3678*^*G4215*^ (*emc2B*) and *CG32441*^*MB09773*^ (*emc10*), which are considered null mutants (Figure S4). We found that different EMC components displayed different functions and importance. Most *emc* null mutants were lethal, except for *emc7* and *emc10* mutants, which had normal levels of Rh1 and normal ERG responses (Table 1 and Figure S7). However, knocking out *emc1, emc2A, emc4* or *emc8/9* in retinal cells using the *Rh1::GFP ey-flp/hid* system led to ablation of retinal cells (Figure S7C and Table 1).

**Table 1:** Summary of *emc* mutants phenotypes

	EMC	Length(aa)	TMD^a^(Y/N)	Lethality	Cell lethality	Rh1 level	PDA
Category 1	EMC1	915	Yes	Lethal	Lethal	NA	NA
	EMC2	282	No	Lethal	Lethal	NA	NA
	EMC4	166	Yes	Lethal	Lethal	NA	NA
	EMC8/9	203	No	Lethal	Lethal	NA	NA
Category 2	EMC6	113	Yes	Lethal	Viable^b^	Decrease	No PDA
	EMC3	247	Yes	Lethal	Viable	Decrease	No PDA
	EMC5	109	Yes	Lethal	Viable	Decrease	No PDA
Category 3	EMC7	245	Yes	Viable	Viable	Normal	Normal
	EMC10	227	Yes	Viable	Viable	Normal	Normal

^a^Transmembrane domain

^b^*emc6* mutation leads to a rapid degeneration in retinal cells

To further demonstrate that different EMC subunits have distinct molecular functions, we performed proteomic analysis of *emc* mutants from different phenotypic categories. Because null *emc2A* and *emc4* mutations completely lacked retinal cells, we generated weak alleles by expressing RNAis of *emc2A, emc4*, or *emc5* in compound eyes using the *GMR-Gal4* driver [28]. The efficiency of *emc2A, emc4*, and *emc5* knockdown was >60% (Figure S8A), and eye morphologies were normal. Importantly, Rh1 levels were significantly reduced in each of these mutants, confirming that these RNAis reduced the function of EMC2A, EMC4, and EMC5, respectively (Figure S8B). These data also demonstrated that EMC2A and EMC4 are required for Rh1 biogenesis. For EMC7, we used null mutants for proteomic analyses. We dissected retinas from control, *emc2A*^*RNAi*^, *emc4*^*RNAi*^, *emc5*^*RNAi*^ and *emc7*^*1*^ flies for the proteomic assay (Fig. 4b). A total of 6,390 proteins were identified, and compared with controls, 50, 44, 39, and 61 proteins were reduced > 50% in *emc2A, emc4*, *emc5*, and *emc7* mutants, respectively (Supplementary Tables 1–4 and Supplementary data). Importantly, a majority of these proteins contained transmembrane domain/signal peptide, and Rh1 levels were reduced in *emc2A, emc4*, and *emc5*, but not *emc7* samples, validating the quantitative proteomic experiments.

Among the reduced proteins, 13 were downregulated in all 4 groups, suggesting that the EMC subunits function cooperatively in the biogenesis of some membrane proteins. In contrast, 16, 4, 9, and 30 downregulated proteins were specific for *emc2A, emc4*, *emc5*, and *emc7* mutants, respectively, indicating potentially distinct functions for individual EMC subunits. Most proteins were downregulated in 2 or 3 genotypes (*emc2A/emc4*/*emc5*: 23, *emc2A/emc4*/*emc7:* 17, *emc2A/emc5*/*emc7*: 14, *emc4*/*emc5/emc7*: 17; *emc2A/emc4*: 29, *emc2A/emc5*: 25, *emc2A/emc7*: 21, *emc4/emc5*: 28, *emc4/emc7*: 27, and *emc5/emc7*: 18) (Fig. 4c and Supplementary Tables 1–4). Together, these data suggest that in addition to functioning as a complex, distinct EMC subunits may be required for the biogenesis of specific membrane proteins.

### Mutations in *emc3, emc5*, and *emc6* lead to retinal degeneration

**Fig. 5:**
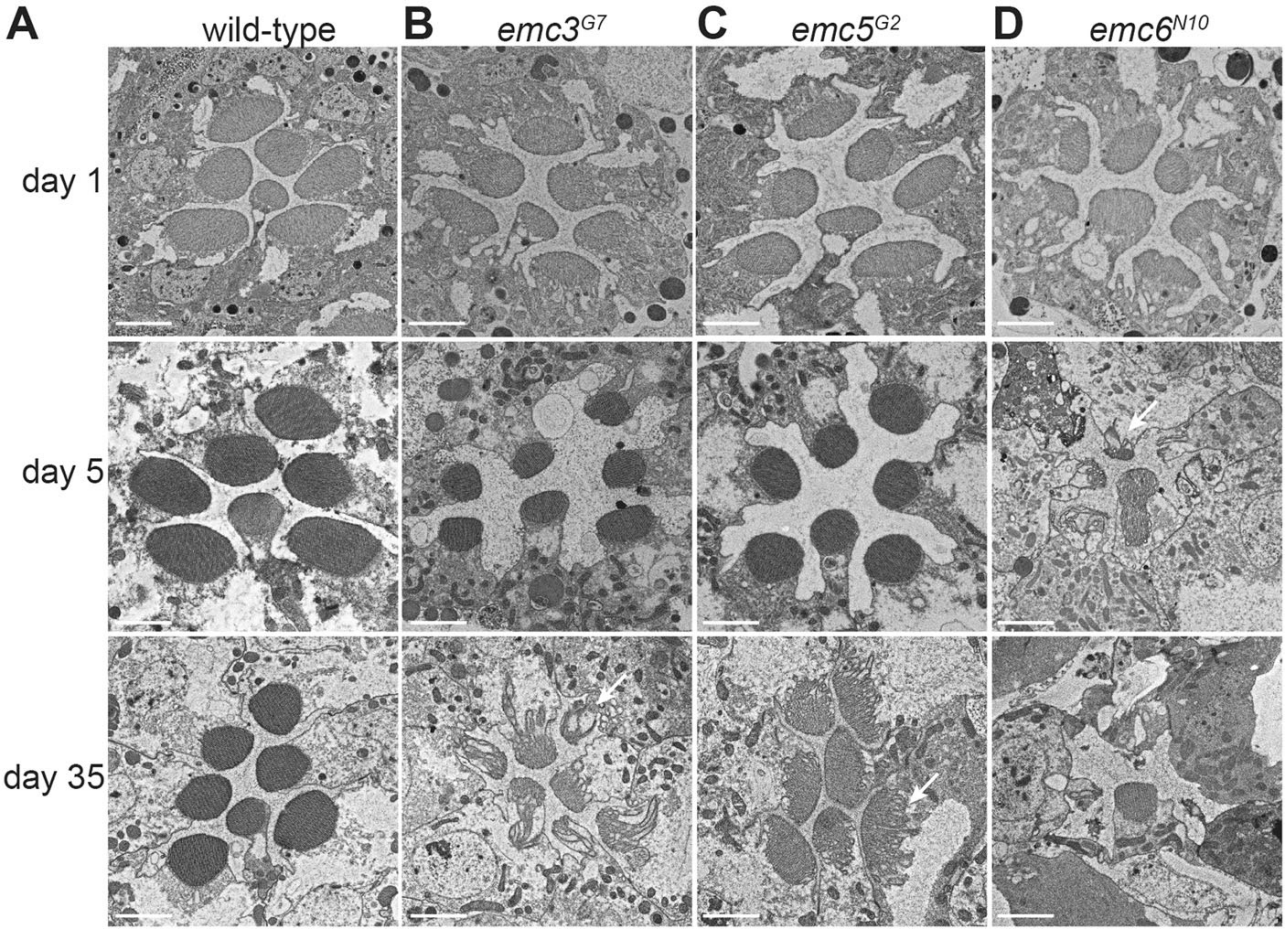
Retinal degeneration in *emc3*, *emc5*, and *emc6* mutant photoreceptors. TEM images of representative ommatidia from (**a**) wild-type, (**b**) *emc3*^*G7*^, (**c**) *emc5*^*G2*^, and (**d**) *emc6*^*N10*^ flies. Flies were raised in a 12h-light/12h-dark (L/D) cycle for indicated time points. The degenerating photoreceptor cells are indicated by arrows. Scale bars are 2 μm

Because mutations in *emc3*, *emc5*, and *emc6* largely reduced Rh1 protein levels and disrupted phototransduction, we examined if photoreceptor integrity depends on these genes. Wild-type ommatidia consistently exhibit seven photoreceptor cells with intact rhabdomeres (Fig. 5a). Young (~1 day old) *emc5*^*G2*^, *emc3*^*G7*^, and *emc6*^*N10*^ mutants exhibited normal retinal morphology with all 7 rhabdomeres, although the rhabdomeres were smaller in *emc6*^*N10*^ flies, reflecting an early stage of retinal degeneration. Both *emc5*^*G2*^ and *emc3*^*G7*^ mutants contained intact rhabdomeres and photoreceptor cells at 5 days of age, whereas 35-day-old flies exhibited retinal degeneration with prominent vacuoles and obvious loss of rhabdomeres (Figs. 5b, c). Thirty-five-day-old *emc5*^*G2*^ and *emc3*^*G7*^ flies exhibited a decrease in ERG amplitude (Figure S9), also reflecting mild retinal degeneration associated with *emc5*^*G2*^ and *emc3*^*G7*^ flies. Consistent with previous results, more severe photoreceptor cell death was associated with *emc6*^*N10*^ mutant flies, which exhibited disruption of rhabdomeres and the accumulation of abnormal vesicles shortly after eclosion (Fig. 5d). These results demonstrated that EMC3, EMC5, and EMC6 are required for photoreceptor cell survival.

### Loss of *Emc3* in mammalian cone cells leads to cone-photoreceptor defects

**Fig. 6:**
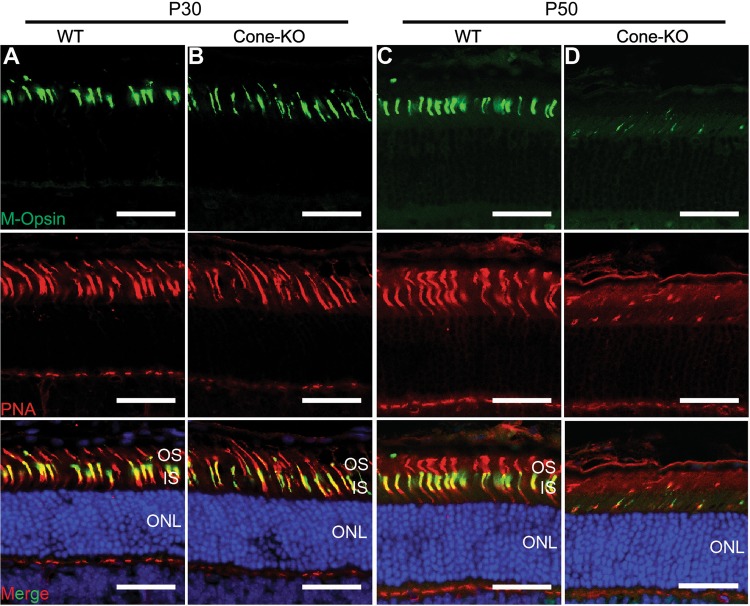
Loss of *Emc3* in mammalian cone cells led to cone cell death. **a**, **b** Retina cryosections from (**a**) WT and (**b**) cone-KO littermate mice at P30. Sections were immunostained for M-Opsin (green) and the cone marker, peanut agglutinin (PNA, red). Cone cell number did not decrease at P30. **c**, **d** Retina cryosections from (**c**) WT and (**d**) cone-KO mice at P50. Sections were immunostained for M-Opsin (green) and PNA (red). There were almost no cone cells in cone-KO retinas. Nuclei were counterstained with DAPI. *ONL* outer nuclear layer, *OS* outer segment, *IS* inner segment. Scale bar is 25 μm

We next assessed the functions of EMC in mammalian photoreceptor cells. A conditional knockout allele of *Emc3* was generated using the CRISPR/Cas9 system (Figure S10). In mouse retina, EMC3 is mainly localized to the outer segment (OS), similar to Rho (Figure S11). Weak staining was also observed in the inner segment of photoreceptor cells (Figure S11). One possible explanation of EMC3 localization to OS might be binding of EMC3 to OS targeted membrane proteins, similar to interaction of EMC3 with ABCA3 in the lung [22]. To investigate the role of *Emc3* in photoreceptor cells, we generated a cone-photoreceptor knockout line using *HRGP-Cre* mice (*Emc3*^*loxp/loxp*^ *HRGP-Cre*: *Emc3* cone-KO) in which Cre is driven by the human red/green pigment gene promoter [34]. *HRGP-Cre* mice were used as controls (WT). In *Emc3* cone-KO retinas, M-opsin levels were not affected on postnatal day 30 (P30) (Figs. 6a, b). However, by P50 very little M-opsin protein could be detected in *Emc3* cone-KO retina sections, suggesting that EMC3 is important for M-opsin stability (Figs. 6c, d).

### Deletion of *Emc3* in adult animals leads to rod cell degeneration

To further assess the role of EMC3 in mature photoreceptor cells, we used tamoxifen-activated CAG-CreER to remove *Emc3* in adult mice. We generated *Emc3*^*loxp/loxp*^; CAG-CreER (named *Emc3* iKO) mice and treated the mutant and control (CAG-CreER or *Emc3*^*loxp/loxp*^) animals with tamoxifen at P20 and assessed the retina 7 and 11 days later (P27 and P31). Tamoxifen induction efficiently deleted *Emc3* in all retinal cells, and EMC3 levels were reduced by 70% at P27 compared with control retinas (Figure S11 and S12).

**Fig. 7:**
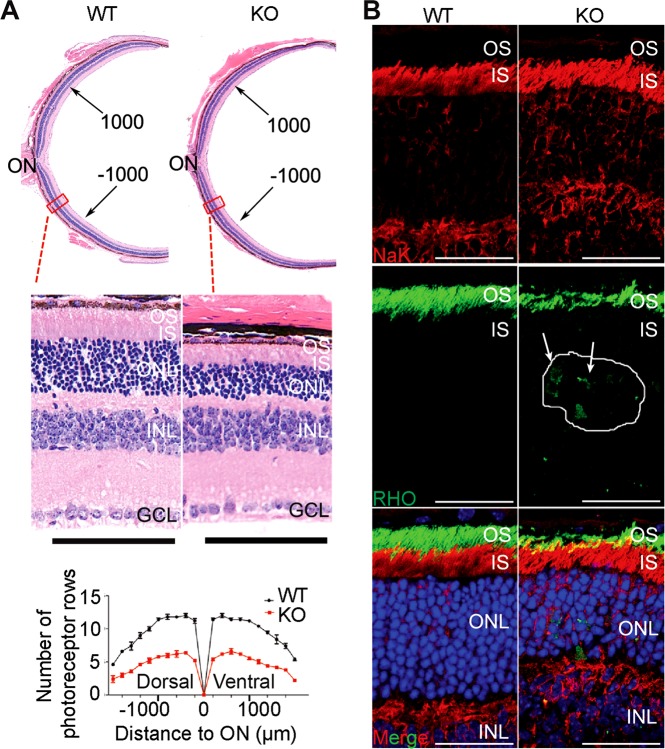
Retinal degeneration and RHO mislocalization in *Emc3* inducible KO mice. **a** Paraffin sections of mice retinas 11 days after induction (P31). Sections were stained with H&E, and quantification of outer nuclear layer is shown on the left. Scale bar is 50 μm. H&E staining of paraffin sections and quantification of outer nuclear layer (ONL) nuclei revealed that the mutant ONL was reduced by ~50% compared to controls 11 days after induction. On top is an illustration of the whole mouse retina. **b** Immunofluorescence labeling of retina cryosections for control (WT) and mutant (KO) littermates at P31. Sections were labeled using antibodies against RHO (green) and DAPI (blue). NaK ATPase antibodies were used to label the inner segment. RHO was mislocalized to the inner segment and cell bodies in KO retinas (arrows). Scale bar is 25 μm

We examined H&E stained retinal sections to assess the morphology of mutant retinas at P27 and P31. At P27, no obvious differences were observed between the mutant and control mice (Figures S13). At P31, however, a thinner outer nuclear layer was seen in the mutant retina (Fig. 7a). The mutant outer nuclear layer (ONL) was reduced by approximately 50% compared with controls, indicating degeneration of Rod photoreceptor cells (Fig. 7a). To investigate if the severe reduction in OS length in *Emc3* KO retinas was caused by defective RHO biosynthesis, we examined retina sections via immunohistochemistry using antibodies against RHO. In control retinas, RHO localized exclusively to the OS. In iKO retinas, RHO levels were reduced, and RHO could be detected in the inner segment and in cell bodies at P27 (Figure S13) and P31 (Fig. 7b). These data suggest that RHO trafficking to the OS was compromised in *Emc3* iKO retinas.

## Discussion

Although EMCs have been implicated in a variety of physiological functions, their molecular function has remained unexplored. EMC components were first identified based on their ability to interact with ERAD components, suggesting that EMC is required for protein folding and/or ER-associated degradation [11, 12]. Later, mutations to EMC subunits were shown to reduce expression of acetylcholine receptors in worms, and rhodopsin in flies, supporting a role for EMC in quality control for a number of multipass transmembrane proteins [24, 47]. Although rhodopsin levels are much lower in *emc3*, *emc5*, and *emc6* mutant animals, rhodopsin is still transported to rhabdomeres, and the mature, deglycosylated form of rhodopsin is primarily detected, indicating that rhodopsin is folded properly in the ER and transported to the plasma membrane. Our data suggest that EMC functions during the de novo biosynthesis of its client protein, rhodopsin, before the protein-folding procedure.

The ERAD pathway is a specialized protein degradation process that removes proteins that fail to fold appropriately in the ER [48]. If loss of EMC destabilizes unfolded rhodopsin in the ER, and/or EMC is connected to the ERAD pathway, disruption of ERAD would be expected to increase rhodopsin levels. However, loss of *hrd1* failed to increase rhodopsin levels in *emc3*, *emc5*, and *emc6* mutant animals, suggesting that EMC has limited function in the quality control of immature rhodopsin, as reported [24, 49]. Our finding that EMC functions during rhodopsin biosynthesis, rather than during later post-translational modifications, is supported by recent reports that EMC is involved in the initial biogenesis of membrane proteins [13, 14, 50].

Since its first identification, EMC components have been shown to interact with each other in several genetic screens [11, 12, 14, 17], and have been suggested to function together as a unit [19, 21]. However, a genome-wide genetic screen in *C. elegans* found that EMC6 is required for the expression of acetylcholine receptors, whereas other EMC subunits are not [47]. Consistent with this notion that different EMC components have distinct functions, several other studies have indicated that individual EMC subunits functions in autophagy, viral assembly, and tissue repair [16, 51, 52]. In our genome-wide screen for genes that regulate rhodopsin levels, the only EMC components to emerge were *emc3*, *emc5*, and *emc6*. Moreover, by generating an entire EMC KO library, we were able to measure the function and importance of each EMC subunit in different contexts, ranging from animal viability, cellular viability, rhodopsin levels, and phototransduction. Our data revealed that *emc1*, *emc2A, emc4* and *emc8/9* are required to maintain basic cellular functions. This may also explain why *emc1*, *emc2, emc4* and *emc8/9* mutants were not identified in our EMS screen. In contrast, no phenotype was observed in *emc7* and *emc10* null mutants, suggesting a less important role of these two EMC components in cellular functions. The weak effects of *emc7* and *emc10* mutations may be explained by functional redundancy. However, this is unlikely as no other EMC7 or EMC10 homologues can be found in flies based on protein sequence.

In weak *emc2* and *emc4* alleles (expressing RNAis), Rh1 levels were significantly decreased as well as applying *emc5* RNAi, indicating that rhodopsin is a common substrate of EMC complexes. Whereas EMC7 and EMC10 are not required for the EMC complex to participate in rhodopsin biogenesis. Quantitative proteomic analysis to compare changes in protein levels in *emc2*, *emc4*, *emc5*, and *emc7* mutants revealed that 13 proteins were downregulated in all 4 mutants, but most (including rhodopsin) were downregulated in ≤3 *emc* mutants. These data provide additional support that EMC subunits are differentially required for the biogenesis of distinct membrane proteins.

Different EMC subunits have been shown to function together in a number of cellular processes. Compared with *emc5*^*G2*^ and *emc3*^*G7*^ null mutant animals, *emc6*^*N10*^ null mutants exhibited lower levels of rhodopsin and TRP proteins, more disrupted rhabdomere morphology, and more severe retinal degeneration. Moreover, EMC5 and EMC3 localized to the ER, whereas EMC6 was also concentrated in intracellular vesicles. However, EMC6 localized to the ER when co-expressed with EMC5, indicating that EMC6 is a *bona fide* component of the EMC complex, while also performing independent functions outside of the ER. The independent functions of EMC6 may explain its role in the biosynthesis of acetylcholine receptors and in autophagy [16, 47]. In S2 cells, EMC6, but not EMC5 and EMC3, partially co-localized with the late endosome maker Rab7, supporting a role for EMC6 in regulating autophagy.

Both rhodopsin and TRP rely on EMC for their biogenesis. However, the *emc3*, *emc5*, and *emc6* mutant flies only lack the PDA, as seen for *ninaE* mutants. These are consistent with previous reports that low levels of TRP is enough for maintaining phototransduction, suggesting that loss of rhodopsin is the major cause of the defective visual responses [53]. Rhodopsin also plays structural roles in maintaining the morphology of photoreceptor cells, therefore loss of rhodopsin in most *ninaE* alleles results in retinal degeneration [54, 55]. Photoreceptor cell degeneration associated with the *emc* mutants could be explained by a disruption in rhodopsin biosynthesis.

The importance of EMC in various human diseases has emerged in recent years. Mutations in EMC1 are associated with global developmental delay, hypotonia, scoliosis, visual impairment and cerebellar atrophy [56, 57]. In addition, EMC is indispensable for West-Nile-Virus-induced cell death [21], and EMC1-dependent stabilization plays an essential role in membrane penetration of a non-enveloped virus [51]. We showed that removal of *Emc3* from photoreceptor cells led to their degeneration. Removal of *Emc3* from the adult retina resulted in the accumulation of RHO in the inner segment or in cell bodies, and the death of rods. We did not observe mislocalized cone opsin in *Emc3* mutants. One potential explanation is that *Emc3* is not essential for cone opsin trafficking. To the best of our knowledge, our study provides the first evidence that an ER membrane protein is essential for mammalian photoreceptor structure.

## Supplementary information

Down-regulated proteins identified in emc2A mutant

Down-regulated proteins identified in emc4 mutant

Down-regulated proteins identified in emc5 mutant

Down-regulated proteins identified in emc7 mutant

Supplemental figures 1-13

Dataset 1

Dateset 2
