# Supplementary material for: ER complex proteins are required for rhodopsin biosynthesis and photoreceptor survival in Drosophila and mice
Source: Cell Death Differ. 2019 Jul 1;27(2):646–61. doi: 10.1038/s41418-019-0378-6 (PMC7206144; doi:10.1038/s41418-019-0378-6)
Supplement: Supplementary file 2 — Down-regulated proteins identified in emc4 mutant [file 41418_2019_378_MOESM2_ESM.docx]

| **Table S2. Down-regulated proteins identified in *emc4* mutant.** | | | |
| --- | --- | --- | --- |
| Gene | GO_Biological function | Signal peptide or transmembrane domain | hit in other *emc* mutants |
| DptB | immune response | N-terminal signal peptide | *emc2A/emc5/emc7* |
| AttA | immune response | N-terminal signal peptide | *emc2A/emc5/emc7* |
| TotC | stress response | N-terminal signal peptide | *emc2A/emc5/emc7* |
| TotA | stress response | N-terminal signal peptide | *emc2A/emc5/emc7* |
| CG4757 | - | N-terminal signal peptide | *emc2A/emc5/emc7* |
| lcs | - | N-terminal signal peptide | *emc2A/emc5/emc7* |
| CG31712 | - | C-terminal transmembrane domain | *emc2A/emc5/emc7* |
| hui | wing disc development | N-terminal signal peptide | *emc2A/emc5/emc7* |
| Vago | defense response to virus | N-terminal signal peptide | *emc2A/emc5/emc7* |
| CG15784 | - | no transmembrane domain | *emc2A/emc5/emc7* |
| CG6484 | transmembrane transport | N-terminal signal peptide | *emc2A/emc5/emc7* |
| CG14329 | - | N-terminal signal peptide | *emc2A/emc5/emc7* |
| CG12763 | immune response | N-terminal signal peptide | *emc2A/emc5/emc7* |
| ninaE | G protein coupled receptor | multiple transmembrane domain | *emc2A/emc5* |
| CG12895 | mitochondrial electron transport | - | *emc2A/emc5* |
| CG9689 | - | N-terminal signal peptide | *emc2A/emc5* |
| trp | calcium ion transmembrane transport | multiple transmembrane domain | *emc2A/emc5* |
| Trpgamma | calcium ion transmembrane transport | multiple transmembrane domain | *emc2A/emc5* |
| CG9377 | proteolysis | N-terminal signal peptide | *emc2A/emc5* |
| inaF-B | response to light stimulus | transmembrane domain | *emc2A/emc5* |
| Bace | proteolysis | N-terminal signal peptide | *emc2A/emc5* |
| Arc1 | vesicle mediated intercellular transport | N-terminal signal peptide | *emc2A/emc5* |
| CecC | immune response | N-terminal signal peptide | *emc2A/emc5* |
| AttC | immune response | N-terminal signal peptide | *emc2A/emc7* |
| Cyp6a14 | oxidationreduction process | N-terminal signal peptide | *emc2A/emc7* |
| RNaseX25 | RNA catabolic process | N-terminal signal peptide | *emc2A/emc7* |
| CG5945 | - | N-terminal signal peptide | *emc2A/emc7* |
| CG9733 | proteolysis | N-terminal signal peptide | *emc5/emc7* |
| Gnmt | regulation of gluconeogenesis | no transmembrane domain | *emc5/emc7* |
| CG10126 | - | transmembrane domain | *emc5/emc7* |
| CG34227 | - | N-terminal signal peptide | *emc5/emc7* |
| DUBAI | protein deubiquitination | transmembrane domain | *emc2A* |
| CG16713 | - | N-terminal signal peptide | *emc2A* |
| Listericin | immune response | N-terminal signal peptide | *emc5* |
| PGRP-SD | immune response | N-terminal signal peptide | *emc7* |
| CG31205 | proteolysis | N-terminal signal peptide | *emc7* |
| Ent1 | nucleoside transport | multiple transmembrane domain | *emc7* |
| CG32486 | - | - | *emc7* |
| CG18067 | multicellular organism reproduction | N-terminal signal peptide | *emc7* |
| cry | circadian rhythm | multiple transmembrane domain | *emc7* |
| CG43317 | - | N-terminal signal peptide |  |
| CG6675 | lipid catabolic process | multiple transmembrane domain |  |
| CG7536 | - | multiple transmembrane domain |  |
| CG5791 | - | N-terminal signal peptide |  |
