# Supplementary material for: ER complex proteins are required for rhodopsin biosynthesis and photoreceptor survival in Drosophila and mice
Source: Cell Death Differ. 2019 Jul 1;27(2):646–61. doi: 10.1038/s41418-019-0378-6 (PMC7206144; doi:10.1038/s41418-019-0378-6)
Supplement: Supplementary file 3 — Down-regulated proteins identified in emc5 mutant [file 41418_2019_378_MOESM3_ESM.docx]

| **Table S3. Down-regulated proteins identified in *emc5* mutant.** | | | |
| --- | --- | --- | --- |
| Gene | GO_Biological function | Signal peptide or transmembrane domain | hit in other *emc* mutants |
| DptB | immune response | N-terminal signal peptide | *emc2A/emc4/emc7* |
| AttA | immune response | N-terminal signal peptide | *emc2A/emc4/emc7* |
| TotC | stress response | N-terminal signal peptide | *emc2A/emc4/emc7* |
| TotA | stress response | N-terminal signal peptide | *emc2A/emc4/emc7* |
| CG4757 | - | N-terminal signal peptide | *emc2A/emc4/emc7* |
| lcs | - | N-terminal signal peptide | *emc2A/emc4/emc7* |
| CG31712 | - | C-terminal transmembrane domain | *emc2A/emc4/emc7* |
| hui | wing disc development | N-terminal signal peptide | *emc2A/emc4/emc7* |
| Vago | defense response to virus | N-terminal signal peptide | *emc2A/emc4/emc7* |
| CG15784 | - | no transmembrane domain | *emc2A/emc4/emc7* |
| CG6484 | transmembrane transport | N-terminal signal peptide | *emc2A/emc4/emc7* |
| CG14329 | - | N-terminal signal peptide | *emc2A/emc4/emc7* |
| CG12763 | immune response | N-terminal signal peptide | *emc2A/emc4/emc7* |
| ninaE | G protein coupled receptor | multiple transmembrane domain | *emc2A/emc4* |
| CG12895 | mitochondrial electron transport | - | *emc2A/emc4* |
| CG9689 | - | N-terminal signal peptide | *emc2A/emc4* |
| trp | calcium ion transmembrane transport | multiple transmembrane domain | *emc2A/emc4* |
| Trpgamma | calcium ion transmembrane transport | multiple transmembrane domain | *emc2A/emc4* |
| CG9377 | proteolysis | N-terminal signal peptide | *emc2A/emc4* |
| inaF-B | response to light stimulus | transmembrane domain | *emc2A/emc4* |
| Bace | proteolysis | N-terminal signal peptide | *emc2A/emc4* |
| Arc1 | vesicle mediated intercellular transport | N-terminal signal peptide | *emc2A/emc4* |
| CecC | immune response | N-terminal signal peptide | *emc2A/emc4* |
| CG11539 | histone acetylation | no transmembrane domain | *emc2A/emc7* |
| CG9733 | proteolysis | N-terminal signal peptide | *emc4/emc7* |
| Gnmt | regulation of gluconeogenesis | no transmembrane domain | *emc4/emc7* |
| CG10126 | - | transmembrane domain | *emc4/emc7* |
| CG34227 | - | N-terminal signal peptide | *emc4/emc7* |
| COX7C | mitochondrial electron transport | - | *emc2A* |
| Listericin | immune response | N-terminal signal peptide | *emc4* |
| scpr-A | multicellular organism reproduction | N-terminal signal peptide |  |
| CG34310 | - | N-terminal signal peptide |  |
| kcc | transmembrane transport | multiple transmembrane domain |  |
| Ent3 | nucleoside transport | multiple transmembrane domain |  |
| CG17249 | mitotic cell cycle checkpoint | no transmembrane domain |  |
| Zasp66 | myofibril assembly | no transmembrane domain |  |
| ect | tube development | N-terminal signal peptide |  |
| CG8550 | proteolysis | N-terminal signal peptide |  |
| CG4935 | mRNA splicing | no transmembrane domain |  |
