# Supplementary material for: ER complex proteins are required for rhodopsin biosynthesis and photoreceptor survival in Drosophila and mice
Source: Cell Death Differ. 2019 Jul 1;27(2):646–61. doi: 10.1038/s41418-019-0378-6 (PMC7206144; doi:10.1038/s41418-019-0378-6)
Supplement: Supplementary file 4 — Down-regulated proteins identified in emc7 mutant [file 41418_2019_378_MOESM4_ESM.docx]

| **Table S4. Down-regulated proteins identified in *emc7* mutant.** | | | |
| --- | --- | --- | --- |
| Gene | GO_Biological function | Signal peptide or transmembrane domain | hit in other *emc* mutants |
| DptB | immune response | N-terminal signal peptide | *emc2A/emc4/emc5* |
| AttA | immune response | N-terminal signal peptide | *emc2A/emc4/emc5* |
| TotC | stress response | N-terminal signal peptide | *emc2A/emc4/emc5* |
| TotA | stress response | N-terminal signal peptide | *emc2A/emc4/emc5* |
| CG4757 | - | N-terminal signal peptide | *emc2A/emc4/emc5* |
| lcs | - | N-terminal signal peptide | *emc2A/emc4/emc5* |
| CG31712 | - | C-terminal transmembrane domain | *emc2A/emc4/emc5* |
| hui | wing disc development | N-terminal signal peptide | *emc2A/emc4/emc5* |
| Vago | defense response to virus | N-terminal signal peptide | *emc2A/emc4/emc5* |
| CG15784 | - | no transmembrane domain | *emc2A/emc4/emc5* |
| CG6484 | transmembrane transport | N-terminal signal peptide | *emc2A/emc4/emc5* |
| CG14329 | - | N-terminal signal peptide | *emc2A/emc4/emc5* |
| CG12763 | immune response | N-terminal signal peptide | *emc2A/emc4/emc5* |
| AttC | immune response | N-terminal signal peptide | *emc2A/emc4* |
| Cyp6a14 | oxidationreduction process | N-terminal signal peptide | *emc2A/emc4* |
| RNaseX25 | RNA catabolic process | N-terminal signal peptide | *emc2A/emc4* |
| CG5945 | - | N-terminal signal peptide | *emc2A/emc4* |
| CG11539 | histone acetylation | no transmembrane domain | *emc2A/emc5* |
| CG9733 | proteolysis | N-terminal signal peptide | *emc4/emc5* |
| Gnmt | regulation of gluconeogenesis | no transmembrane domain | *emc4/emc5* |
| CG10126 | - | transmembrane domain | *emc4/emc5* |
| CG34227 | - | N-terminal signal peptide | *emc4/emc5* |
| Bet1 | vesicle mediated transport | C-terminal transmembrane domain | *emc2A* |
| Tsp42En | cell surface receptor | multiple transmembrane domain | *emc2A* |
| Tsp42Eo | cell surface receptor | multiple transmembrane domain | *emc2A* |
| PGRP-SD | immune response | N-terminal signal peptide | *emc4* |
| CG31205 | proteolysis | N-terminal signal peptide | *emc4* |
| Ent1 | nucleoside transport | multiple transmembrane domain | *emc4* |
| CG32486 | - | - | *emc4* |
| CG18067 | multicellular organism reproduction | N-terminal signal peptide | *emc4* |
| cry | circadian rhythm | multiple transmembrane domain | *emc4* |
| EbpIII | response to virus | N-terminal signal peptide |  |
| w | eye pigment metabolic process | multiple transmembrane domain |  |
| RabX5 | Rab protein signal transduction | no transmembrane domain |  |
| CG14512 | oligosaccharide biosynthetic process | no transmembrane domain |  |
| ATPCL | citrate metabolic process | - |  |
| CG5196 | protein palmitoylation | multiple transmembrane domain |  |
| CG17322 | UDPglucose metabolic process | multiple transmembrane domain |  |
| Als2 | regulation of GTPase activity | - |  |
| Pitslre | protein phosphorylation | - |  |
| yellow-d2 | cuticle pigmentation | N-terminal signal peptide |  |
| Amy-p | carbohydrate metabolic process | N-terminal signal peptide |  |
| fs(1)M3 | eggshell formation | N-terminal signal peptide |  |
| CG17807 | tRNA methylation | - |  |
| IMPPP | immune response | N-terminal signal peptide |  |
| SLC22A | transmembrane transport | multiple transmembrane domain |  |
| CG8993 | cell redox homeostasis | no transmembrane domain |  |
| bw | transmembrane transport | multiple transmembrane domain |  |
| CG14419 | - | N-terminal signal peptide |  |
| arg | arginine catabolic process to ornithine | no transmembrane domain |  |
| ppk26 | sodium ion transport | multiple transmembrane domain |  |
| Nek2 | protein phosphorylation | no transmembrane domain |  |
| CG30423 | multivesicular body sorting pathway | multiple transmembrane domain |  |
| CG16772 | - | N-terminal signal peptide |  |
| CG8303 | wax biosynthetic process | multiple transmembrane domain |  |
| CG11825 | copper ion homeostasis | transmembrane domain |  |
| CG7299 | - | N-terminal signal peptide |  |
| GstZ1 | glutathione metabolic process | no transmembrane domain |  |
| Lsp1alpha | - | N-terminal signal peptide |  |
| GluRIB | cation transport | multiple transmembrane domain |  |
| GNBP-like3 | response to fungus | N-terminal signal peptide |  |
