# Supplementary material for: ER complex proteins are required for rhodopsin biosynthesis and photoreceptor survival in Drosophila and mice
Source: Cell Death Differ. 2019 Jul 1;27(2):646–61. doi: 10.1038/s41418-019-0378-6 (PMC7206144; doi:10.1038/s41418-019-0378-6)
Supplement: Supplementary file 5 — Supplemental figures 1-13 [file 41418_2019_378_MOESM5_ESM.pdf]

Supplemental Figure 1

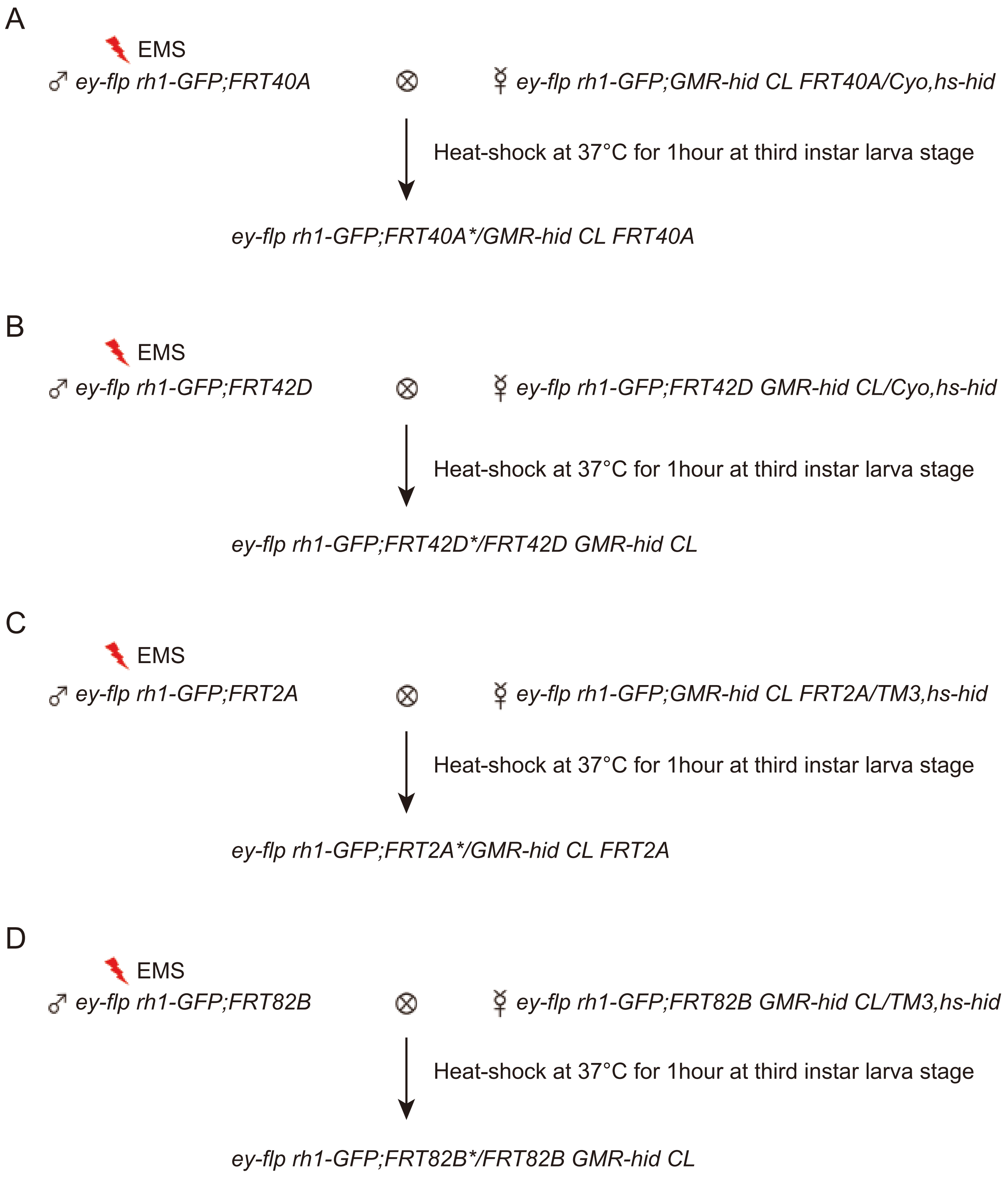

Figure S1. Strategy for F1 screening for recessive mutations. The (A) *ey-flp,Rh1-GFP;FRT40A*, (B) *ey-flp,Rh1-GFP;FRT42D*, (C) *ey-flp,Rh1-GFP;FRT2A* and (D) *ey-flp,Rh1-GFP;FRT82B* flies were isogenized and flies were mutagenized by feeding with 25 mM EMS (Sigma) in 2% sucrose for 8 h before being mated to (A) *ey-flp Rh1-GFP;GMR-hid CL FRT40A/Cyo hs-hid*, (B) *ey-flp Rh1-GFP;FRT42D GMR-hid CL/Cyo hs-hid*, (C) *ey-flp Rh1-GFP;GMR-hid CL FRT2A/TM3 hs-hid* and (D) *ey-flp Rh1-GFP;FRT82B GMR-hid CL/TM3 hs-hid* flies, respectively. Flies were heat shocked at 37°C for 1 h to avoid having heterozygous flies among the F1 progeny. Rh1 fluorescence was assessed in F1 flies using a fluorescent stereomicroscope day 1 and 5 following eclosion.

Supplemental Figure 2

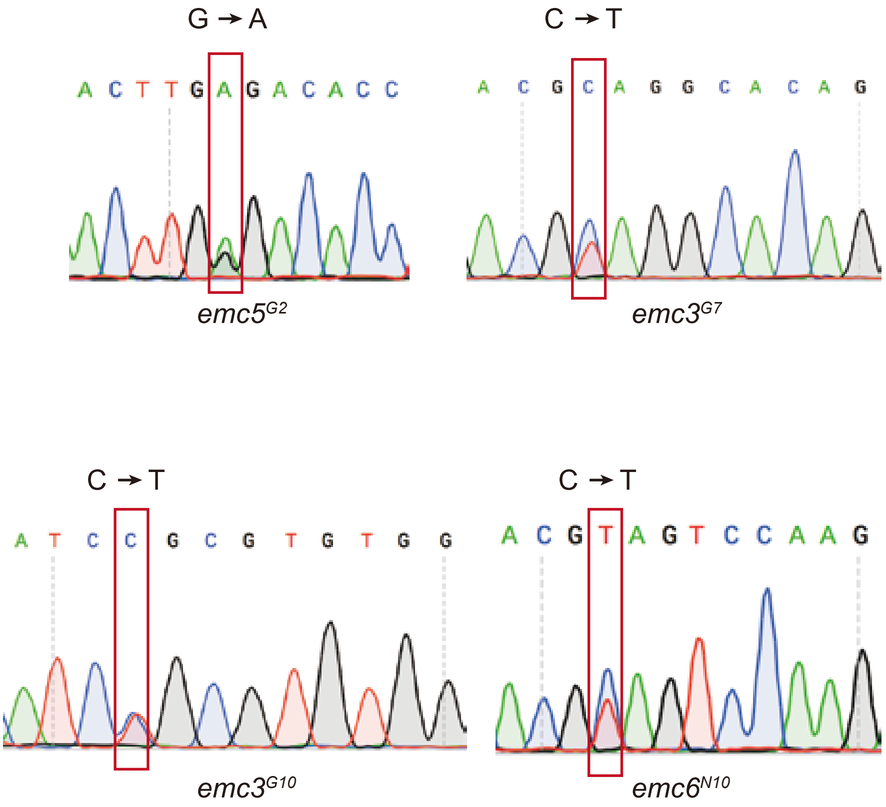

Figure S2. Verification of the *emc3*^{G7}, *emc3*^{G10}, *emc5*^{G2} and *emc6*^{N10} loci by genomic DNA sequencing. PCR products targeting the *emc3*, *emc5* and *emc6* loci were amplified from genomic DNA isolated from *emc3*^{G7}, *emc3*^{G10}, *emc5*^{G2} and *emc6*^{N10} flies, and subsequently sequenced.

Supplemental Figure 3

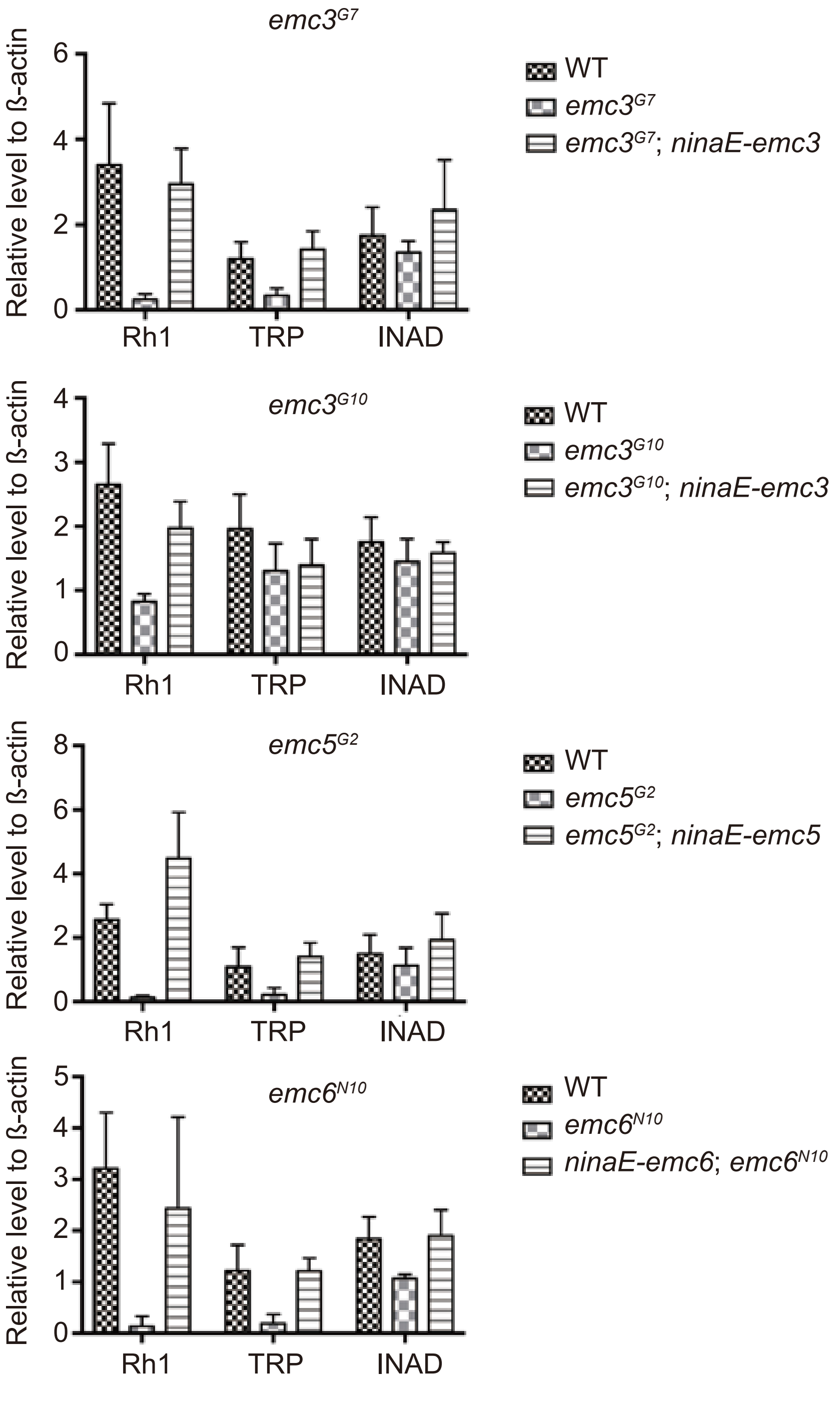

Figure S3. Relative levels of Rh1, TRP, and INAD in *emc3*^{G7}, *emc3*^{G10}, *emc5*^{G2} and *emc6*^{N10} mutants. Rh1, TRP and INAD protein levels in *emc3*^{G7}, *emc3*^{G10}, *emc5*^{G2} and *emc6*^{N10} head extracts were compared with the band intensity obtained from wild-type extracts. ß-actin served as the loading control. Quantification was performed using a LI-COR Odyssey imaging system. SDs are indicated.

Supplemental Figure 4

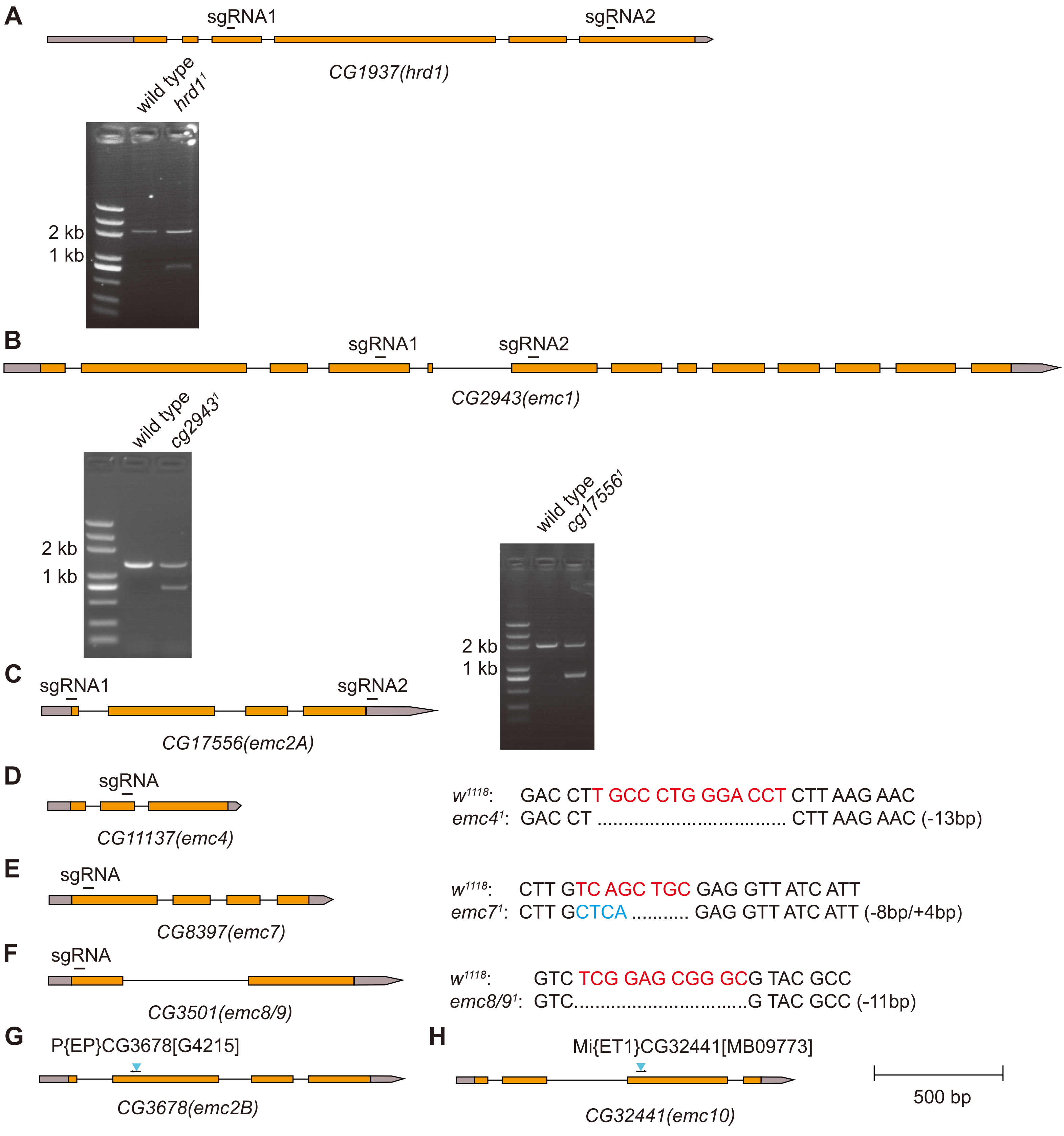

Figure S4. Generation of *hrd1¹*, *emc1¹*, *emc2A¹*, *emc4¹*, *emc7¹*, *emc8/9¹*, *emc2B^G4215^* and *emc10^MB09773^* flies. Schematic for gene knock-out by sgRNA targeting. Organization of the *hrd1*, *emc1 (CG2943)*, *emc2A (CG17556)*, *emc4 (CG11137)*, *emc7 (CG8397)*, *emc8/9 (CG3501)*, *emc2B (CG3678)*, and *emc10 (CG32441)* loci are shown. Boxes represent exons with the coding region filled orange. sgRNA primer pairs were used to generate the (A) *hrd1¹*, (B) *emc1¹* and (C) *emc2A¹* alleles, and PCR products obtained from *hrd1¹*, *emc1¹* and *emc2A¹* mutants showed successful gene deletions. A single sgRNA primer was used to generate the (D) *emc4¹*, (E) *emc7¹* and (F) *emc8/9¹* mutations, which caused a frame-shift. The *emc4¹*, *emc7¹* and *emc8/9¹* loci were verified by DNA sequencing. The (G) *emc2B^G4215^* and (H) *emc10^MB09773^* mutants were obtained by P-element inserted in the coding region.

Supplemental Figure 5

A

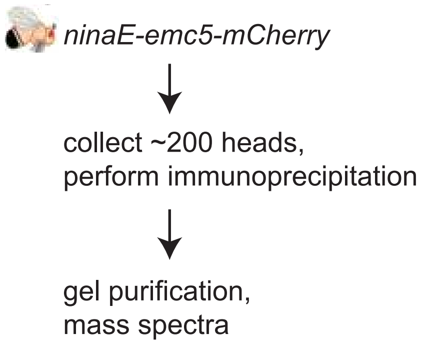

B

	EMC5	
	trial1	trial2
EMC1		
EMC2		
EMC3	2	1
EMC4	1	3
EMC5	5	6
EMC6		
EMC7	4	5
EMC8/9		4
EMC10	3	2

Figure S5. Interaction among EMC subunits. (A) Flow diagram of the experiment for identifying EMC5 interacting proteins by mass spectra after co-immunoprecipitation with mCherry-tagged EMC5. (B) EMC subunits identified from two independent mass spectra trials, ranked by their mascot scores.

Supplemental Figure 6

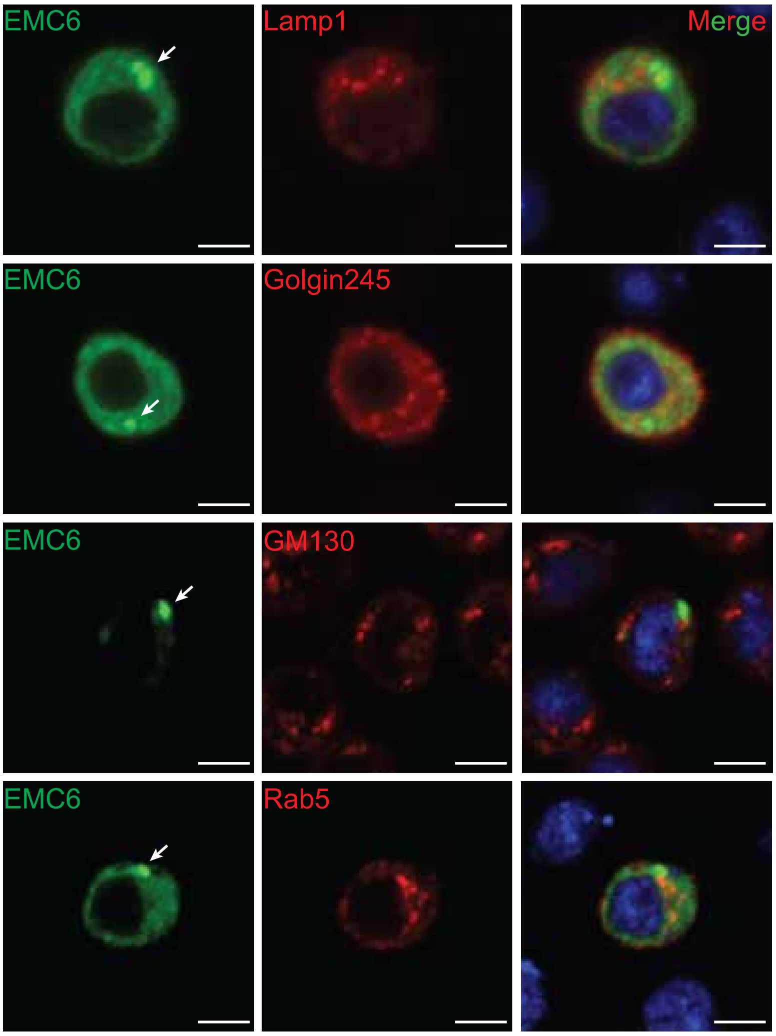

Figure S6. Localization of EMC6. S2 cells were transiently transfected with emc6-GFP, together with intracellular compartment markers: lamp1-RFP (lysosome marker), golgin245-RFP (trans-Golgi marker), or rab5-RFP (early endosome marker), or staindeds with GM130 (cis-Golgi marker). EMC6 puncta are indicated by arrows. Scale bar is 5 μm.

Supplemental Figure 7

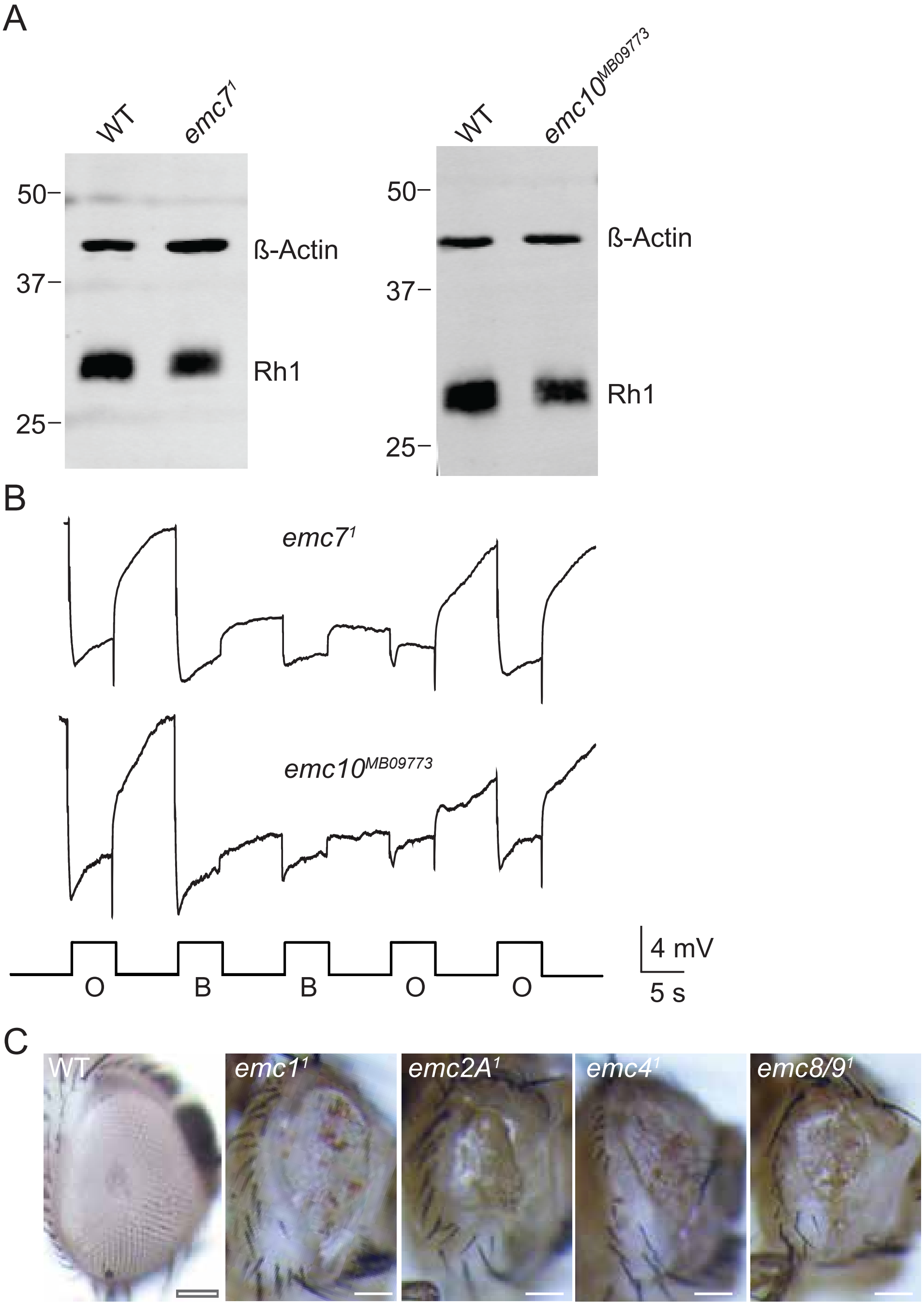

Figure S7. *emc1¹*, *emc2A¹*, *emc4¹*, *emc7¹*, *emc8/9¹*, and *emc10^{MB09773}* mutant flies show different phenotypes. (A) Rhodopsin levels were not affected in *emc7¹* and *emc10^{MB09773}* flies. Protein extracts from 1/4 head of each genotype (1 d after eclosion) were loaded, and were probed with antibodies against Rh1, ß-actin. (B) ERG recordings from both *emc7¹* and *emc10^{MB09773}* flies show wild-type PDA when induced by blue (B) light. (C) Homozygous mutations of *emc1¹* (*ey-flp rh1-GFP;FRT82B emc1¹ /FRT82B GMR-hid CL*), *emc2A¹*(*ey-flp rh1-GFP;FRT82B emc2A¹/FRT82B GMR-hid CL*), *emc4¹* (*ey-flp rh1-GFP;emc4¹ FRT80B/GMR-hid CL FRT80B*) and *emc8/9¹* (*ey-flp rh1-GFP;FRT42D emc8/9¹ /FRT42D GMR-hid CL*) caused severe retinal cell death, scale bar is 100 μm.

Supplemental Figure 8

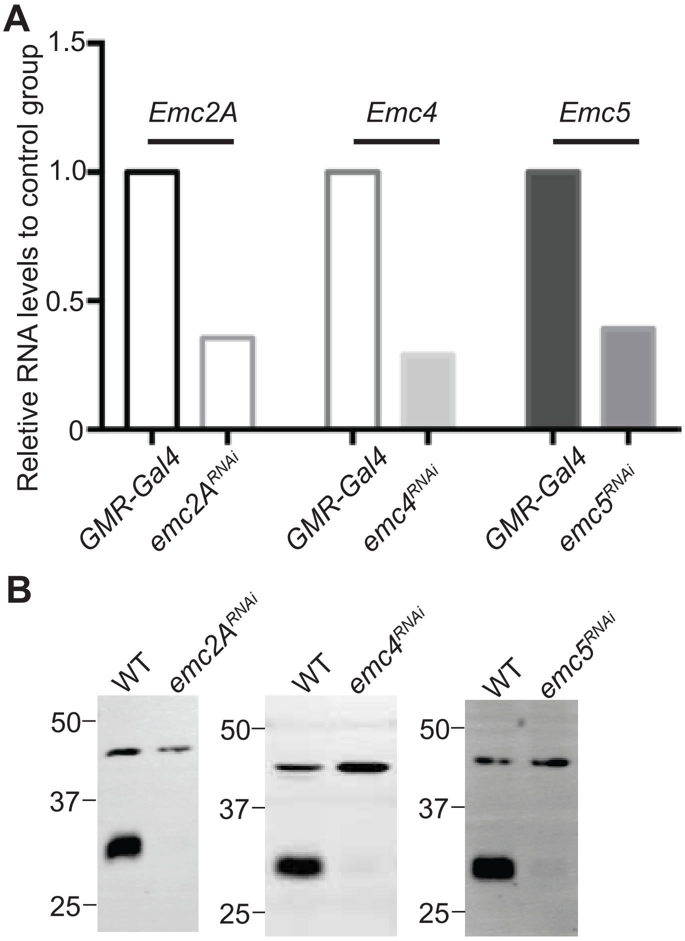

Figure S8. Verification of *emc2A*^{RNAi}, *emc4*^{RNAi} and *emc5*^{RNAi} flies. (A) RNAi efficiency was determined by using quantitative Real Time PCR (qPCR). Total RNA was extracted from isolated fly retina of wild type (*GMR-Gal4/+*), *emc2A*^{RNAi} (*GMR-Gal4/emc2A*^{RNAi}), *emc4*^{RNAi} (*GMR-Gal4/emc4*^{RNAi}), and *emc5*^{RNAi} (*GMR-Gal4/emc5*^{RNAi}), and RP49 was served as control. (B) Rhodopsin levels were reduced in *emc2A*^{RNAi}, *emc4*^{RNAi}, and *emc5*^{RNAi} flies. Protein extracts from 1/4 head of each genotype (1 d after eclosion) were loaded, and were probed with antibodies against Rh1 and ß-actin antibodies.

Supplemental Figure 9

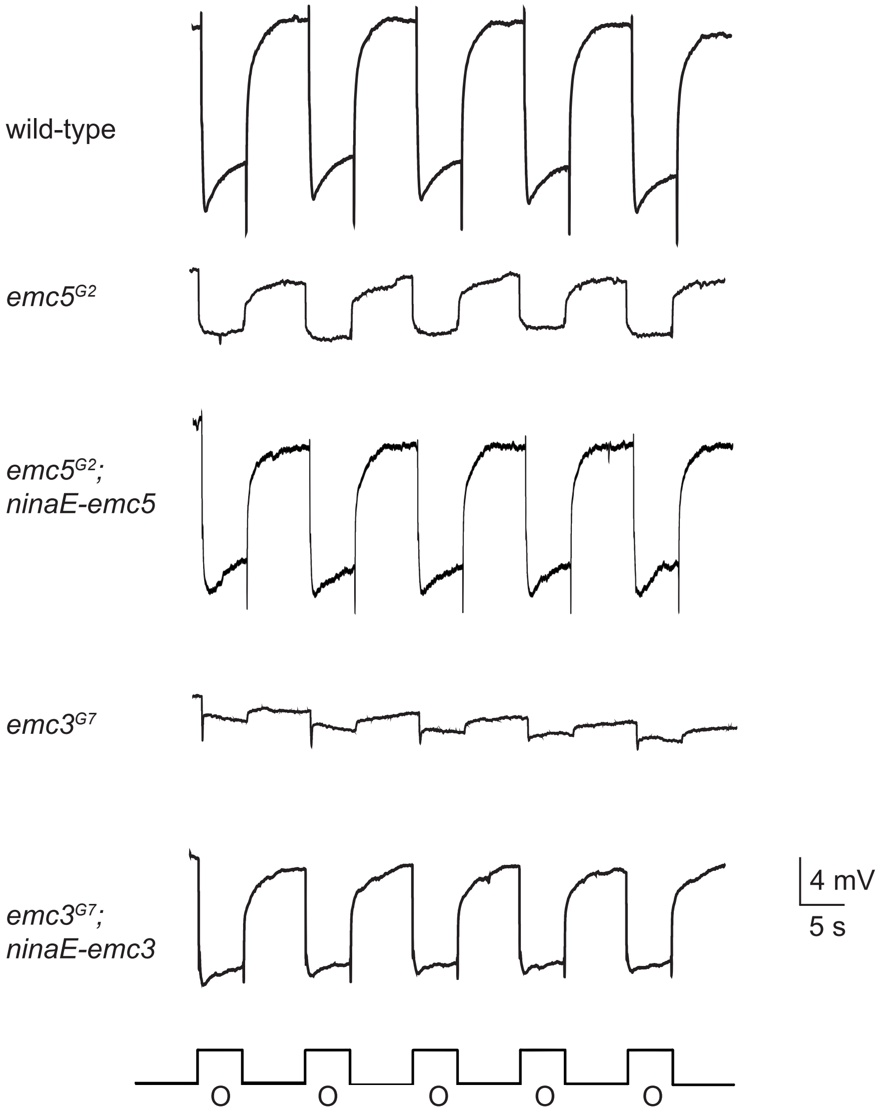

Figure S9. ERG response was reduced in 35-day-old *emc3* and *emc5* mutants. ERG amplitudes from 35-day-old *emc3*^{G7} and *emc5*^{G2} mutant flies were significantly lower than 35-day-old wild-type flies. ERG Amplitudes were restored in *emc3*^{G7}*;ninaE-emc3* and *emc5*^{G2}*;ninaE-emc5* flies. Flies were dark-adapted for 2 min and subsequently exposed to 5-s pulses of orange (O) light. At least 10 flies for each genotype have been tested. ERG were recorded from 35-day-old flies maintained under 12-h light/12-h dark cycle.

Supplemental Figure 10

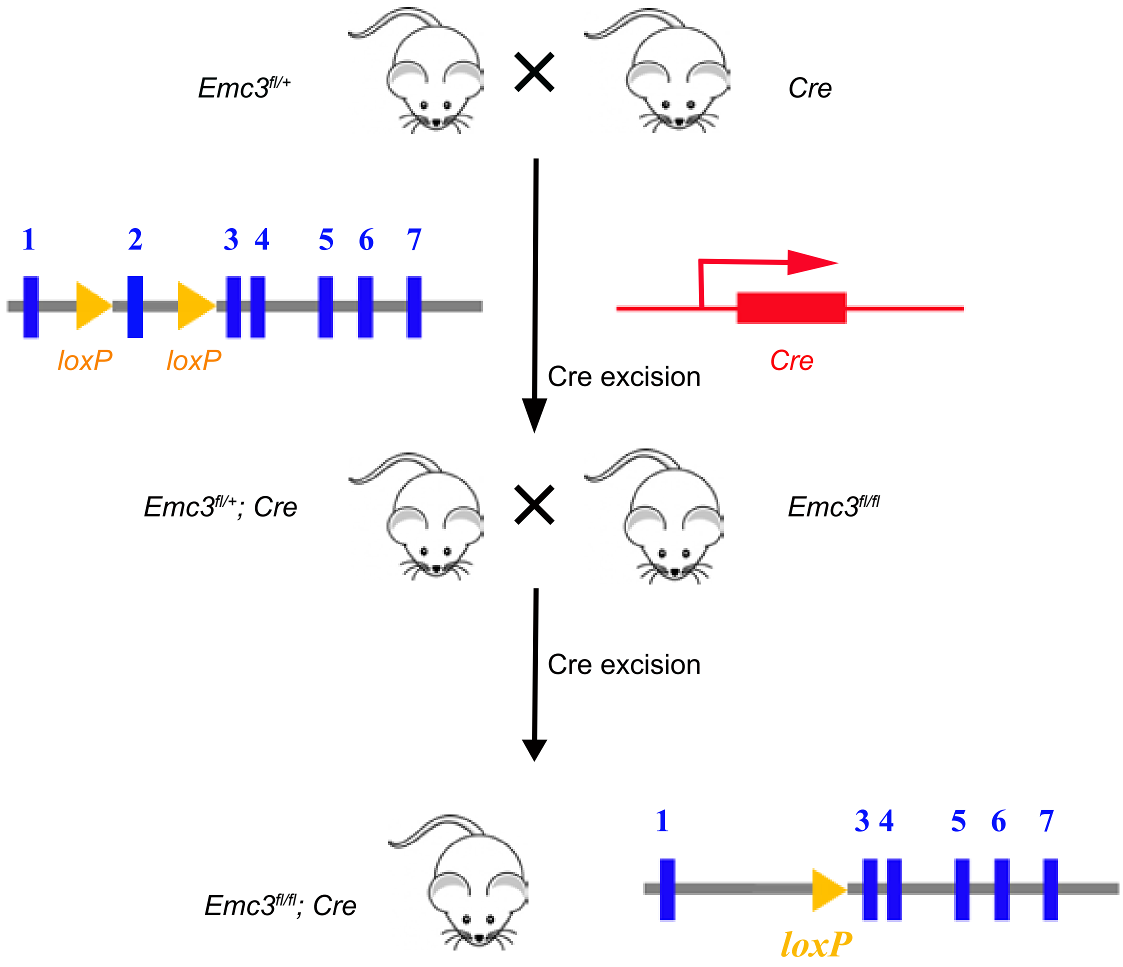

Figure S10. Schematic diagram of *Emc3* conditional knockout. Conditional deletion of *Emc3* with *HRGP-Cre* or *CAG-Cre*. Design of the *Emc3* conditional knock-out allele (cKO) is shown. The critical exon 2 is flanked by loxP sites. The *Emc3* cKO allele was crossed to *HRGP-Cre* or *CAG-Cre*.

Supplemental Figure 11

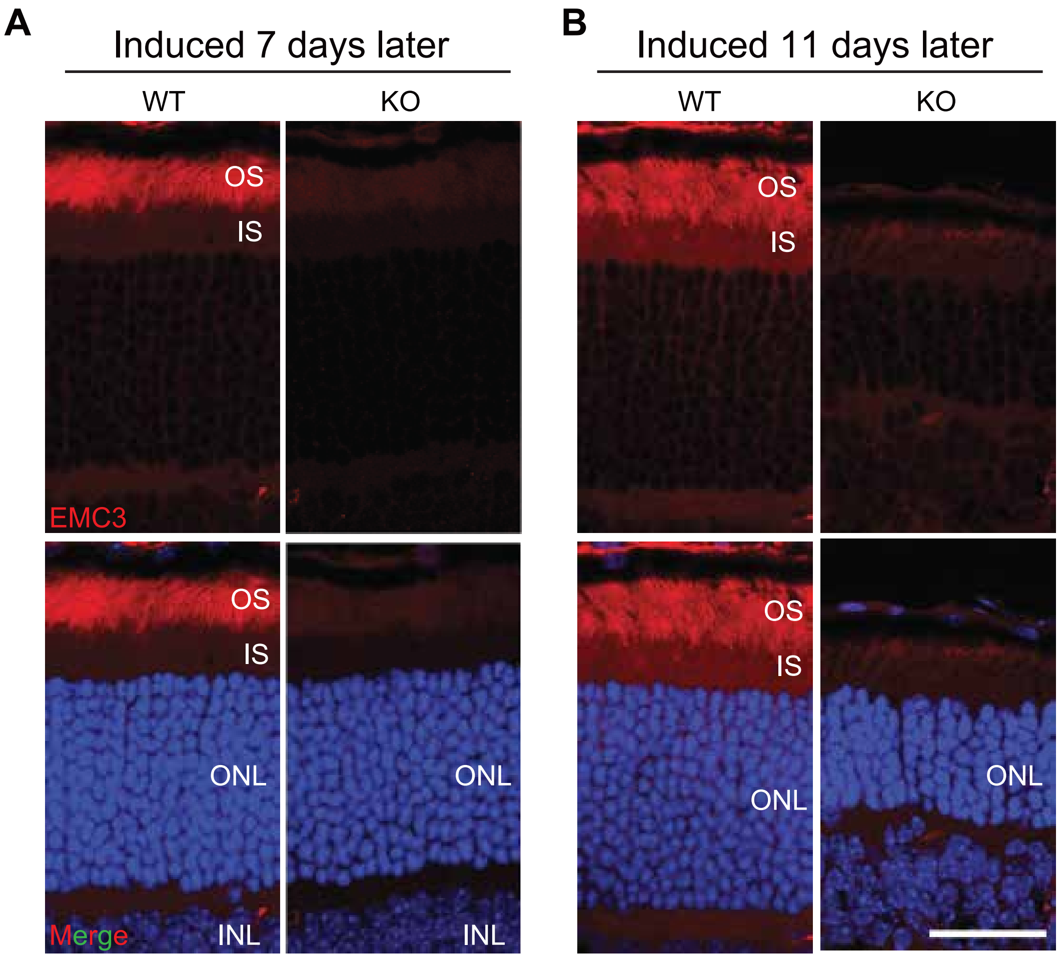

Figure S11. Removal of *Emc3* in the retina reduced levels of EMC3 in the retinas of *Emc3* inducible knockout (iKO) animals. (A-B) Immunofluorescence analysis showed that EMC3 levels (red) were dramatically decreased in iKO mouse retinas 7 and 11 days following tamoxifen induction. DAPI (blue) was used to stain the nuclei. OS, outer segment; IS, inner segment; ONL, outer nuclear layer; INL, inner nuclear layer. Scale bar is 20 μm.

Supplemental Figure 12

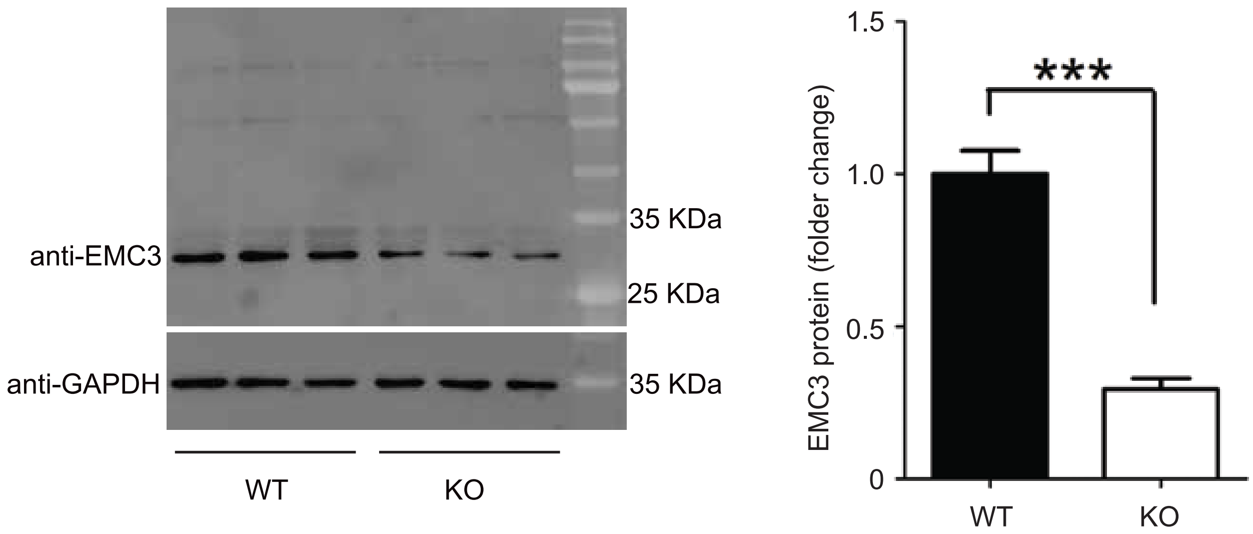

Figure S12. Removal *Emc3* in the retina led to diminished Emc3 expression in the retinas of *Emc3* inducible knockout (KO). Western blot analysis showed EMC3 expression decreased 70% in KO mouse retina after induced with tamoxifen 7 days later. GAPDH was used as loading control. ***, P<0.001. P values refer to comparison of *Emc3* KO vs. WT (wild type), by two-tailed t-test.

Supplemental Figure 13

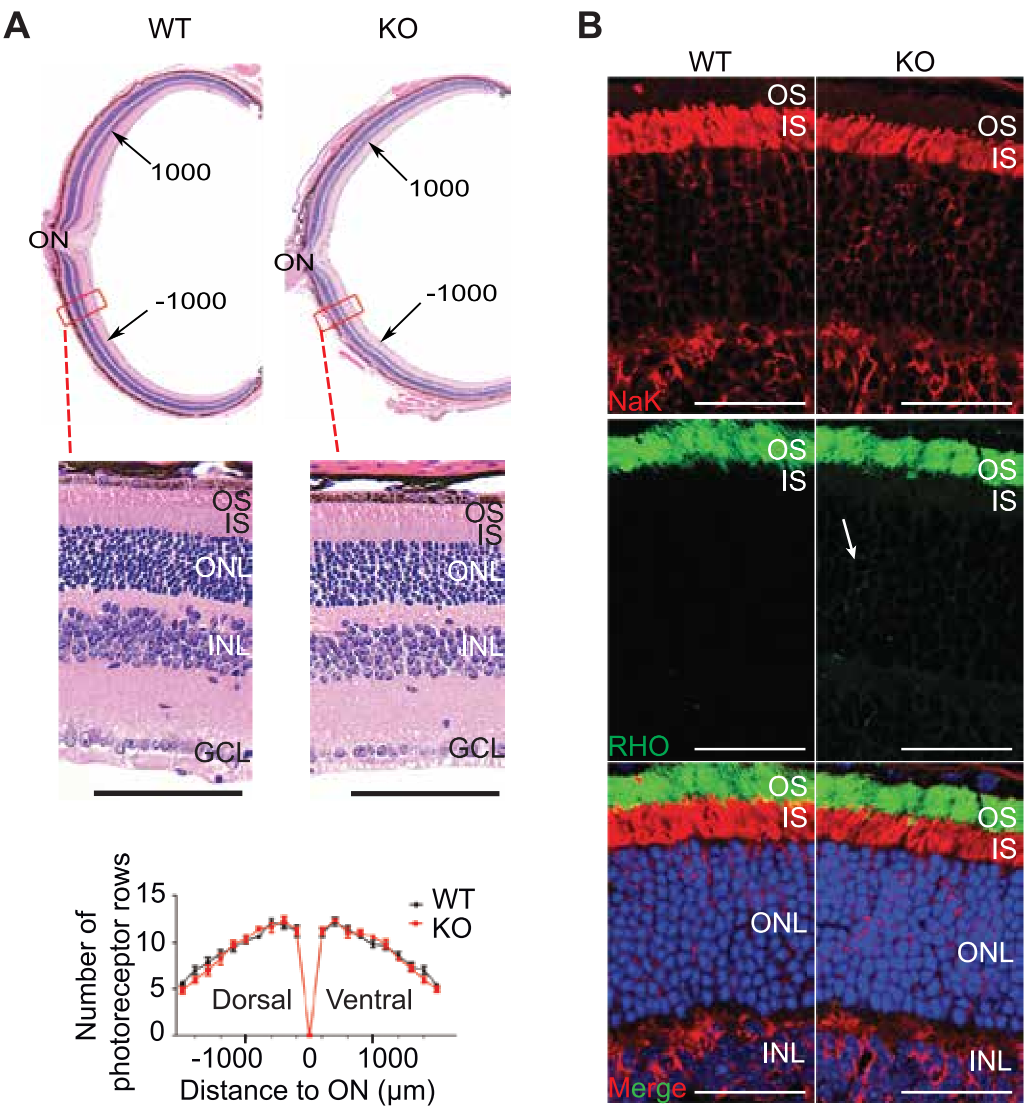

Figure S13. Retinal degeneration and RHO mislocalization in *Emc3* inducible KO mice. (A) Paraffin sections of mice retinas 7 days after induction (P27). Sections were stained with H&E, and quantification of outer nuclear layer is shown on the left. Scale bar is 50 μm. (B) Immunofluorescence labeling of retina cryosections for control (WT) and mutant (KO) littermates at P27. Sections were labeled using antibodies against RHO (green) and DAPI (blue). NaK ATPase antibodies were used to label the inner segment. RHO was mislocalized to outer nuclear layer. OS, outer segment; IS, inner segment; ONL, outer nuclear layer; INL, inner nuclear layer. Scale bar, 20 μm.
